# Implementing Bogoliubov Transformations Beyond the Shale–Stinespring Condition

**DOI:** 10.1007/s10955-025-03415-y

**Published:** 2025-03-25

**Authors:** Sascha Lill

**Affiliations:** Dipartimento di Matematica, Università degli Studi di Milano, Via Cesare Saldini 50, 20133 Milan, Italy

**Keywords:** Non-perturbative renormalization, Dressing transformations, Infinite tensor product spaces, Fock space extensions, Bogoliubov transformations, Quadratic Hamiltonians

## Abstract

We define infinite tensor product spaces that extend Fock space, and allow for implementing Bogoliubov transformations which violate the Shale or Shale–Stinespring condition. So an implementation on the usual Fock space would not be possible. Both the bosonic and fermionic case are covered. Conditions for implementability in an extended sense are stated and proved. From these, we derive conditions for a quadratic Hamiltonian to be diagonalizable by a Bogoliubov transformation that is implementable in the extended sense. We apply our results to Bogoliubov transformations from quadratic bosonic interactions and BCS models, where the Shale or Shale–Stinespring condition is violated, but an extended implementation nevertheless works.

## Introduction

In quantum many-body systems and non-perturbative quantum field theory (QFT), one often encounters situations in which a formal expression *H* for a Hamiltonian is given in the physics literature, but can a priori not be interpreted as a self-adjoint operator that generates dynamics on a Hilbert space. Over the past decades, a plethora of mathematical tools has been conceived to overcome this issue [1, 2].

An established but little investigated tool in this area are Fock space extensions, such as the infinite tensor product (ITP) framework, introduced by von Neumann [3]. There have been attempts to apply this framework to quantum electrodynamics (QED) scattering theory [4–6]. Rigorous results exist on the implementation of Weyl transformations (i.e., linear exponents) that are not implementable on the usual Fock space [7–9].

The present paper takes the step from linear to quadratic exponents, i.e., from Weyl to Bogoliubov transformations: We derive conditions which ensure that a Bogoliubov transformation, which is not implementable on the usual Fock space, can nevertheless be implemented on a suitable ITP space.

Non-implementable Weyl- and Bogoliubov transformations are both a well-researched topic in the $$ C^*$$-algebraic formulation of quantum dynamics [10]. Therefore, they serve as an ideal test area for mathematical tools that may ultimately turn out advantageous for more sophisticated operator transformations.

Let us explain a bit more precisely, how the implementation of Bogoliubov transformations via ITPs works. Roughly speaking, a Bogoliubov transformation $$ \mathcal {V}= \left( {\begin{smallmatrix} u &  v \\ \overline{v} &  \overline{u} \end{smallmatrix}} \right) $$ replaces annihilation operators $a^\dagger (f), a(f)$ by

$$\begin{aligned} b^\dagger (f) = a^\dagger (u f) + a(v \overline{f}), \quad b(f) = a^\dagger (v \overline{f}) + a(u f), \end{aligned}$$

with *f* being an element of the one-particle Hilbert space $\mathfrak {h}$, where *u*, *v* are linear operators on $\mathfrak {h}$ and where $\overline{f}$ is the complex conjugate of *f*. This replacement may diagonalize quadratic Hamltonians *H* [11–14]. Related transformations allow for eliminating inconvenient terms of higher order in more sophisticated *H* [15–18].

It is desirable to find a unitary operator $\mathbb {U}_\mathcal {V}$ on Fock space $\mathscr {F}$, such that $\mathbb {U}_\mathcal {V}$ establishes the replacement $a^\sharp \mapsto b^\sharp$ via

1 $$\begin{aligned} \mathbb {U}_\mathcal {V}a^\dagger (f) \mathbb {U}_\mathcal {V}^* = b^\dagger (f), \quad \mathbb {U}_\mathcal {V}a(f) \mathbb {U}_\mathcal {V}^* = b(f). \end{aligned}$$

In that case we say that $\mathbb {U}_\mathcal {V}$ implements the transformation $\mathcal {V}$ and we call $\mathcal {V}$ “implementable” (in the regular sense). It is well-known [19, 20] that $\mathcal {V}$ is implementable, if and only if the **Shale condition** (bosonic case) or the **Shale–Stinespring condition** (fermionic case) holds, which asserts that ${\textrm{tr}}(v^*v) < \infty$. Situations with non-implementable Bogoliubov transformations occur, for instance, in relativistic models [21, 22], and within many-body systems of infinite size [23–25].

We prove that under certain conditions, $\mathcal {V}$ is nevertheless implementable on certain ITP spaces $\widehat{\mathscr {H}} = \prod _{k \in \mathbb {N}}^\otimes \mathscr {H}_k$, which extend all of $\mathscr {F}$, while being a sum of typically uncountably many spaces that are naturally isomorphic to $\mathscr {F}$. We further elucidate the structure of $\widehat{\mathscr {H}}$ in the end of Sect. 2.2, as well as in Appendix A.

**Fig. 1:**
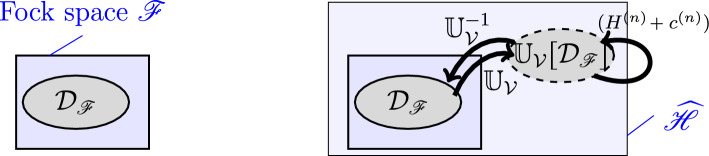
Renormalization of *H* using ITPs

Also, the way how we diagonalize formal Hamiltonians *H* by implementers $\mathbb {U}_\mathcal {V}$ differs from the usual procedure on Fock space: For some formal *H* consisting of a product of $a^\dagger$- and *a*-operators, we aim at defining (see Fig. 1)

2 $$\begin{aligned} \widetilde{H} = \mathbb {U}_\mathcal {V}^{-1} (H + c) \mathbb {U}_\mathcal {V} \end{aligned}$$

on a dense subspace $\mathcal {D}_\mathscr {F}\subseteq \mathscr {F}$, where $\widetilde{H}$ is the version of *H* with $a^\sharp$ replaced by $b^\sharp$ and with normal ordering applied. The “renormalization constant” *c* stems from normal ordering and can be infinite. While (2) may not always be achieved, we can still split $H = \sum _{n \in \mathbb {N}} H^{(n)}$ and $c = \sum _{n \in \mathbb {N}} c^{(n)}$, such that

3 $$\begin{aligned} \widetilde{H} = \sum _{n \in \mathbb {N}} \mathbb {U}_\mathcal {V}^{-1} \left( H^{(n)} + c^{(n)}\right) \mathbb {U}_\mathcal {V}. \end{aligned}$$

That is, we define an operator $\mathbb {U}_\mathcal {V}: \mathcal {D}_\mathscr {F}\rightarrow \widehat{\mathscr {H}}$ such that $(H^{(n)} + c^{(n)})$ maps the space $\mathbb {U}_\mathcal {V}[\mathcal {D}_\mathscr {F}] \subset \widehat{\mathscr {H}}$ into itself. Further, $\widetilde{H}$ allows for a self-adjoint extension and can thus be seen as the well-defined renormalized version of *H*.

Our **main result** is that in the extended sense, specified in Definition 5.1, $\mathcal {V}$ can indeed be implemented on $\widehat{\mathscr {H}}$ in the bosonic (Theorem 5.5) and the fermionic case (Theorem 5.6) if the spectrum of the operator $v^*v$ is countable. We may then naturally extend the implementer to a unitary operator $\mathbb {U}_\mathcal {V}: \widehat{\mathscr {H}} \rightarrow \widehat{\mathscr {H}}$, see Remark 5.7.

It is worth mentioning that within the **extended state space** (ESS) framework [26], a similar result was recently proven even for arbitrary $v^*v$ in the bosonic case [27]. In Appendix B, we briefly discuss a much simpler ESS construction for $v^*v$ having discrete spectrum, which allows for an extended implementation in both the bosonic and fermionic case (Propositions B.3 and B.4).

With respect to ITPs, the fermionic ESS implementation requires the additional restriction to a finite number of particle–hole transformed modes. Nevertheless, we expect a fermionic ESS construction, similar to [27], to also be successful for generic $v^*v$.

By contrast, the ITP construction cannot be expected to achieve an implementation for generic $v^*v$: Still, the ITP space would be well-defined as $\prod _{x \in X}^\otimes \mathscr {H}_x$ with *X* being a possibly uncountable set related to $\sigma (v^*v)$. However, $a^*(f) = \sum _x f(x) a^*_x$ would only be defined for countable sums in *x*. So $a^*(f)$ would be ill-defined unless *f*(*x*) is everywhere 0 apart from countably many *x*.

The ultimate goal would be to implement more general operator transformations *W* such that

4 $$\begin{aligned} \widetilde{H} = W^{-1} (H + c) W, \end{aligned}$$

is well-defined on $\mathcal {D}_\mathscr {F}\subseteq \mathscr {F}$ and allows for a self-adjoint extension. Here, *c* is a general counterterm and not necessarily just a constant. Transformations *W* as above arise from non-perturbative cutoff renormalization [28–33], when formally removing the IR- or UV-cutoff from the employed dressing transformations. In contrast to cutoff renormalization, the direct renormalization by Fock space extensions does not involve limiting processes or cutoffs that break Lorentz invariance. A similar cutoff-free non-perturbative renormalization technique, also known as “interior–boundary conditions” (IBC), has recently been proposed and investigated [34–42]. However, IBC renormalization is limited to cases where the free and interacting Hamiltonian can be defined in the same CCR/CAR representation. This can be a severe restriction, for instance, in relativistic models,^1^ and renormalization by Fock space extensions is designed to overcome this issue.

The rest of this paper is structured as follows: In Sect. 2, we give the basic definitions of second quantization and the ITP framework. Section 3 recaps known properties on Bogoliubov transformations that are needed for the extended implementation. In Sect. 4, we define the $\mathcal {V}$-dependent ITP spaces $\widehat{\mathscr {H}}$ and prove that creation and annihilation operators are well-defined on them (Lemma 4.9). On these ITP spaces, we define implementability in Sect. 5, and prove that it is satisfied (Theorems 5.5 and 5.6). Section 6, is devoted to the diagonalization of Hamiltonians in the extended sense (Propositions 6.3 and 6.4). In Sect. 7 we examine two examples for a diagonalization in the extended sense.

## Basic Definitions

### Fock Space Notions

We consider a measure space $(X, \mu )$ with $X \subseteq \mathbb {R}^d$, where we focus on $X = \mathbb {R}^d$ and $X = \mathbb {N}$. A configuration of $N \in \mathbb {N}_0$ particles is given by the tuple $q = (\varvec{x}_1, \ldots , \varvec{x}_N)$, which is an element of the ordered configuration space

5 $$\begin{aligned} \mathcal {Q}(X) := \bigsqcup _{N = 0}^\infty \mathcal {Q}(X)^{(N)} := \bigsqcup _{N = 0}^\infty X^N \;. \end{aligned}$$

$\mathcal {Q}(X)$ allows for a standard topology and a measure $\mu _N$ on each sector $\mathcal {Q}(X)^{(N)}$, hence yielding a topology and a measure $\mu _\mathcal {Q}$ on $\mathcal {Q}(X)$. The full Fock space is then

6 $$\begin{aligned} \mathscr {F}(X) := L^2(\mathcal {Q}(X),\mu _\mathcal {Q}) \;. \end{aligned}$$

The corresponding scalar product is $\langle \Phi , \Psi \rangle := \int _{\mathcal {Q}(X)} \overline{\Phi (q)} \Psi (q) \; {\textrm{d}}q$ with $\Phi , \Psi \in \mathscr {F}(X)$ and the overline denoting complex conjugation. For $\Psi \in C_0(\mathcal {Q}(X))$, a unique continuous representative function exists. This includes smooth functions $\Psi \in C^\infty (\mathcal {Q}(X))$ and smooth functions with compact support $\Psi \in C_c^\infty (\mathcal {Q}(X))$.

For describing bosonic/fermionic particle exchange symmetries, we introduce the symmetrization operators $S_+, S_-: \mathscr {F}(X) \rightarrow \mathscr {F}(X)$ defined by

7 $$\begin{aligned} (S_\pm \Psi )(\varvec{x}_1, \ldots , \varvec{x}_N) := \frac{1}{N!} \sum _{\sigma \in S_N} (\pm 1)^{(1-{\textrm{sgn}}(\sigma ))/2} \Psi (\varvec{x}_{\sigma (1)}, \ldots , \varvec{x}_{\sigma (N)}) \;, \end{aligned}$$

with permutation group $S_N$. Here, $(1-{\textrm{sgn}}(\sigma ))/2$ is 0 if the permutation is even, and 1 if it is odd. The bosonic ($+$) and fermionic ($-$) Fock space is given by

8 $$\begin{aligned} \mathscr {F}_\pm (X) := S_\pm [\mathscr {F}(X)]. \end{aligned}$$

The (*N*) -sectors of Fock space are $\mathscr {F}(X)^{(N)}:= L^2(\mathcal {Q}(X)^{(N)}, \mathbb {C})$, with symmetrized and antisymmetrized analogues $\mathscr {F}_\pm ^{(N)}$. Note that we may equivalently write

9 $$\begin{aligned} \mathscr {F}(X) := \bigoplus _{N = 0}^\infty \mathscr {F}(X)^{(N)} \;, \quad \mathscr {F}(X)^{(N)} := \underbrace{\mathfrak {h}\otimes \ldots \otimes \mathfrak {h}}_{N {\text { times}}} \;, \end{aligned}$$

with $\mathfrak {h}:= L^2(X)$. A change of basis then allows us to identify $\mathfrak {h}$ with $\ell ^2 = L^2(\mathbb {N})$ (or a subspace thereof), thus identifying $\mathscr {F}(X)$ with $\mathscr {F}(\mathbb {N})$. In the following, we drop the (*X*) if not explicitly needed.

Creation and annihilation operators $a^\dagger (f), a(f)$ for some $f \in \mathfrak {h}$ can be defined by using $q{\setminus }\varvec{x}_j \in X^{N-1}$ for denoting the removal of one particle $\varvec{x}_j$ from a configuration $q \in X^N$:

10 $$\begin{aligned} \begin{aligned} (a_\pm ^\dagger (f) \Psi )(q)&= \sum _{j = 1}^N \frac{(\pm 1)^j}{\sqrt{N}} f(\varvec{x}_j) \Psi (q \setminus \varvec{x}_j) \;,\\ (a_\pm (f) \Psi )(q)&= \sqrt{N+1} \int \overline{f(\varvec{x})} \Psi (q,\varvec{x}) \; {\textrm{d}}\mu (\varvec{x}) \;. \end{aligned} \end{aligned}$$

It is well-known that the fermionic operators $a_-, a^\dagger _-$ are bounded and hence defined on all $\Psi \in \mathscr {F}_-$, while the bosonic $a_+, a^\dagger _+$ are unbounded, but can still be defined on a dense subspace of $\mathscr {F}_+$. Further, (10) implies the canonical commutation/anticommutation relations (CCR/CAR):

11 $$\begin{aligned} \left[ a_\pm (f),a_\pm ^\dagger (g)\right] _\pm = \langle f,g \rangle _\mathfrak {h}, \quad \left[ a_\pm (f),a_\pm (g)\right] _\pm = 0 = \left[ a_\pm ^\dagger (f),a_\pm ^\dagger (g)\right] _\pm , \end{aligned}$$

with commutator $[A,B]_+ = [A,B] = AB-BA$ and anticommutator $[A,B]_- = \{A,B\} = AB+BA$. In the following, we will drop the indices $\pm$ if there is no risk of confusion.

It is also customary to just consider $a(f), a^\dagger (f)$ not as operators, but as formal expressions within a ^*^ -algebra^2^

12 $$\begin{aligned} \mathcal {A}= \mathcal {A}_\pm \quad {\text {generated by}} \quad \left\{ a_\pm (f), a_\pm ^\dagger (f) \; \mid \; f \in \mathfrak {h}\right\} . \end{aligned}$$

The involution is given by $$ a(f)^* = a^\dagger (f) $$ and the multiplication in $$ \mathcal {A}$$ is such that the CCR/CAR hold. In particular, $$ \mathcal {A}_- $$ is a $$ C^*$$-algebra by boundedness of operators.

### Infinite Tensor Products

In this subsection, we give a quick introduction to general ITPs, as introduced by von Neumann [3], see also [44]. Some useful lemmas and remarks concerning this construction are given in Appendix A. For a more thorough discussion, we refer the reader to [3].

We consider a (possibly uncountable) index set *I*, and for each $k \in I$ a Hilbert space $\mathscr {H}_k$ with scalar product $\langle \cdot , \cdot \rangle _k$ and induced norm $\Vert \cdot \Vert _k$. The aim is to construct a vector space, which is generated formally by ITPs

13 $$\begin{aligned} \Psi = \prod _{k \in I}^\otimes \Psi _k, \end{aligned}$$

or equivalently, by families $(\Psi ) = (\Psi _k)_{k \in I}, \; \Psi _k \in \mathscr {H}_k$. If *I* is countable, then $(\Psi ) = (\Psi _1, \Psi _2, \ldots )$ defines a sequence. Each family gets assigned the formal expression

14 $$\begin{aligned} \Vert (\Psi ) \Vert := \prod _{k \in I} \Vert \Psi _k \Vert _k, \end{aligned}$$

which we will later use for defining a norm. In order to answer the question, whether (14) defines a complex number, one introduces the notions of convergence within a (possibly uncountable) sum:

- For $z_k \in \mathbb {C}, \; k \in I$, we call $\sum _{k \in I} z_k$ or $\prod _{k \in I} z_k$ convergent to $a \in \mathbb {C}$, if for all $\delta > 0$, there exists some finite set $I_\delta$, such that for all finite sets $J \subseteq I$ with $I_\delta \subseteq J$, we have
  15 $$\begin{aligned} \left| a -\sum _{k \in J} z_k \right| \leqslant \delta \quad {\text {or}} \quad \left| a -\prod _{k \in J} z_k \right| \leqslant \delta , \quad {\text {respectively}}. \end{aligned}$$

A simple consequence of this definition is that $\sum _{k \in I} z_k$ can only converge if $z_k \ne 0$ occurs for only countably many $k \in I$. So the question of convergence reduces to that of sequence convergence. Further, it is shown in [3] that $\prod _{k \in I} z_k < \infty$ if and only if we have $z_k = 0$ for at least one $k \in I$ or if $\sum _{k \in I} \vert z_k - 1 \vert < \infty$. The heuristic reason is that $\prod _{k \in I} z_k = \exp {\left( \sum _{k \in I} \ln {z_k} \right) }$ and $\ln {z_k}$ can be linearly approximated near 1 as $\ln {z_k} = 1 - z_k + \mathcal {O}((1-z_k)^2)$.

In case $\prod _{k \in I} |z_k|$ converges to a nonzero number, then $\prod _{k \in I} z_k$ converges if and only if no infinite phase variation occurs. That is, if ${\textrm{arg}}(z_k) \in (-\pi , \pi ]$ is the phase of the complex number $z_k$, then it is required that

16 $$\begin{aligned} \sum _{k \in I} |{\textrm{arg}}(z_k)| < \infty \;. \end{aligned}$$

In order to establish a notion of convergence, even when (16) is violated, one defines that $\prod _{k \in I} z_k$ is *quasi-convergent* if and only if $\prod _{k \in I} |z_k|$ converges. A family $(\Psi ) = (\Psi _k)_{k \in I}$ is now called a

- *C***-sequence** ($(\Psi ) \in {\textrm{Cseq}}$) if and only if $\prod _{k \in I} \Vert \Psi _k \Vert _k < \infty$,
- $C_0$**-sequence** if and only if $\sum _{k \in I} \left| \Vert \Psi _k \Vert _k - 1 \right|< \infty \quad \Leftrightarrow \quad \sum _{k \in I} \left| \Vert \Psi _k \Vert _k^2 - 1 \right| < \infty$.

Each $C_0$-sequence is also a *C*-sequence. For all *C*-sequences, we have a well-defined value $\Vert (\Psi ) \Vert \in \mathbb {C}$ by (14) and each *C*-sequence, that is not a $C_0$-sequence, must automatically satisfy $\Vert (\Psi ) \Vert = 0$. However, (14) only defines a seminorm, since there exist $(\Psi ) \ne 0$ with $\prod _{k \in I} \Vert \Psi _k \Vert _k = 0$. To make it a norm, one defines

as the space of all functionals on ${\textrm{Cseq}}$ which are conjugate-linear in each component.

Following [3], we can embed  by identifying $(\Phi ) \in {\textrm{Cseq}}$ with the functional

17 $$\begin{aligned} \Phi = \iota ((\Phi )): (\Psi ) \mapsto \prod _{k \in I} \langle \Phi _k, \Psi _k \rangle _k \;. \end{aligned}$$

This identification essentially sets up an equivalence relation $\sim _{\textrm{C}}$ on ${\textrm{Cseq}}$, where $(\Phi ) \sim _{\textrm{C}} (\Phi ')$, whenever $\iota ((\Phi )) = \iota ((\Phi '))$. In Proposition A.1, we show that equivalence is given if and only if $(\Phi )$ and $(\Phi ')$ just differ by a family of complex factors $(c_k)_{k \in I}$ with $\prod _{k \in I} c_k = 1$. The functionals in $\iota [{\textrm{Cseq}}]$ are then the equivalence classes and the span of these functionals is denoted by [3]:

- $$ \widetilde{\prod }^{\otimes }_{k \in I} \mathscr {H}_k:= {\textrm{span}}(\iota [{\textrm{Cseq}}])$$.

In the following, we may drop the embedding map $\iota$ and simply identify $(\Phi )$ with $\Phi$, whenever the identification is obvious. An inner product $\langle \cdot , \cdot \rangle$ can uniquely be defined on $\widetilde{\prod }^{\otimes }_{k \in I} \mathscr {H}_k$ via

18 $$\begin{aligned} \langle \Phi , \Psi \rangle = \prod _{k \in I} \langle \Phi _k, \Psi _k \rangle _k \;, \end{aligned}$$

which is a quasi-convergent product that we understand to be 0 whenever it is not convergent. $\langle \cdot , \cdot \rangle$ makes $\widetilde{\prod }^{\otimes }_{k \in I} \mathscr {H}_k$ a pre-Hilbert space and induces a norm $\Vert \Phi \Vert$ agreeing with (14) under identification $\Vert \Phi \Vert = \Vert (\Phi ) \Vert$. This norm allows completing $\widetilde{\prod }^{\otimes }_{k \in I} \mathscr {H}_k$ to a Hilbert space:

- The **infinite tensor product space** $\widehat{\mathscr {H}} = \prod ^{\otimes }_{k \in I} \mathscr {H}_k$ is defined as the space of all , such that there exists a Cauchy sequence $(\Phi ^{(r)})_{r \in \mathbb {N}} \subset \widetilde{\prod }^{\otimes }_{k \in I} \mathscr {H}_k$ with respect to $\Vert \cdot \Vert$ that converges to $\Phi$ in the weak-^*^ topology on .

One may indeed extend $\langle \cdot , \cdot \rangle$ to $\widehat{\mathscr {H}}$, making the latter a Hilbert space [3]. In order to further analyze its structure, we divide $\widehat{\mathscr {H}}$ into subspaces, For which we split the set of $C_0$-sequences into equivalence classes via

- equivalence: $$ (\Phi ) \sim (\Psi ) \quad : \Leftrightarrow \quad \sum _{k \in I} \left| \langle \Phi _k, \Psi _k \rangle - 1 \right| < \infty $$
- weak equivalence: $$ (\Phi ) \sim _w (\Psi ) \quad : \Leftrightarrow \quad \sum _{k \in I} \big \vert |\langle \Phi _k, \Psi _k \rangle | - 1 \big \vert < \infty $$

The respective equivalence classes are called *C* and $C_w$, and the corresponding linear spaces of an equivalence class are

- $$ \prod ^{\otimes C}_{k \in I} \mathscr {H}_k:= \overline{{\textrm{span}}\{ \Psi \; \mid \; \exists (\Psi ) \in C: \iota ((\Psi )) = \Psi \}}^{\Vert \cdot \Vert } $$ for equivalence
- $\prod ^{\otimes C_w}_{k \in I} \mathscr {H}_k:= \overline{{\textrm{span}}\{ \Psi \; \mid \; \exists (\Psi ) \in C_w: \iota ((\Psi )) = \Psi \}}^{\Vert \cdot \Vert }$ for weak equivalence

Now, each $C_0$-sequence $(\Psi )$ in some equivalence class $[(\Omega )] = C$ (with $(\Omega ) \in {\textrm{Cseq}}$ being interpreted as the vacuum vector) can be written in **coordinates** as follows [3, Theorem V]: Choose an orthonormal basis $(e_{k,n})_{n \in \mathbb {N}_0}$ for each $\mathscr {H}_k$, such that $\Omega _k = e_{k,0}$ (we think of $e_{k,0}$ as mode *k* being in the vacuum). Then, $(\Psi ) = (\Psi _k)_{k \in I}$ is uniquely specified by the coordinates $c_{k,n}:= \langle e_{k,n}, \Psi _k \rangle _k \in \mathbb {C}$. In this coordinate representation, it is true that

- $\prod ^{\otimes C}_{k \in I} \mathscr {H}_k$ is the closure of the space spanned by all normalized $C_0$-sequences, where $c_{k,0} = 1$ for all but finitely many $k \in I$.

Or heuristically speaking, “almost all $\Psi _k$ are in the vacuum”. By [3, Theorem V], also a generic $\Psi \in \prod ^{\otimes C}_{k \in I} \mathscr {H}_k$ can be written as

19 $$\begin{aligned} \Psi = \sum _{n(\cdot ) \in F} a(n(\cdot )) \prod ^\otimes _{k \in I} e_{k,n(k)} \;, \end{aligned}$$

with *F* being the countable set of all functions $n:I \rightarrow \mathbb {N}_0$ with $n(k) = 0$ for almost all $k \in I$, and $a(n(\cdot )) \in \mathbb {C}$ being the coordinates of $\Psi$ with $\sum _{n(\cdot ) \in F} |a(n(\cdot ))|^2 < \infty$.

## Bogoliubov Transformations

In this section, we introduce our notation for Bogoliubov transformations and recap some important properties. Standard references on the subject are [45] and [46].

### Transformation on Operators

Consider the one-operator subspace $W_1$ of $\mathcal {A}$, which is linearly spanned by $a_\pm ^\dagger (f), a_\pm (g)$, $f, g \in \mathfrak {h}$. By an algebraic Bogoliubov transformation, we mean any bijective map $\mathcal {V}_A: W_1 \rightarrow W_1$, which sends $a_\pm ^\dagger (f), a_\pm (g)$ to a new set of creation and annihilation operators $b_\pm ^\dagger (f), b_\pm (g)$, such that $b_\pm ^\dagger (f)$ is the adjoint of $b_\pm (f)$ and the CAR/CCR are conserved under $\mathcal {V}_\mathcal {A}$ and its adjoint.

To facilitate the presentation, we fix a basis $(e_j)_{j \in \mathbb {N}} \subset \mathfrak {h}$ which identifies $f \in \mathfrak {h}$ with an equally denoted vector $\varvec{f}= (f_j)_{j \in \mathbb {N}} \in \ell ^2$ by $f_j:= \langle e_j, f \rangle$. This way, we can also identify $a^\dagger (f) + a(\overline{g}) \in W_1$ with a vector $(\varvec{f}, \varvec{g}) \in \ell ^2 \oplus \ell ^2$, so an algebraic Bogoliubov transformation $\mathcal {V}_A$ is identified with a linear operator $\mathcal {V}$ on $\ell ^2 \oplus \ell ^2$, which we just call “Bogoliubov transformation”. We may also encode sums of creation and annihilation operators by vector pairs $(\varvec{f},\varvec{g}) \in \ell ^2 \oplus \ell ^2$ via the generalized creation/annihilation operators

20 $$\begin{aligned} \begin{aligned} A_\pm ^\dagger : \ell ^2 \oplus \ell ^2&\rightarrow \mathcal {A}_\pm \;,&(\varvec{f}_1, \varvec{f}_2)&\mapsto a_\pm ^\dagger (\varvec{f}_1) + a_\pm (\overline{\varvec{f}_2}) = \sum _j \left( f_{1,j} a_\pm ^\dagger (e_j) + f_{2,j} a_\pm (e_j)\right) \;,\\ A_\pm : \ell ^2 \oplus \ell ^2&\rightarrow \mathcal {A}_\pm \;,&(\varvec{g}_1, \varvec{g}_2)&\mapsto a_\pm (\varvec{g}_1) + a_\pm ^\dagger (\overline{\varvec{g}_2}) = \sum _j \left( \overline{g_{1,j}} a_\pm (e_j) + \overline{g_{2,j}} a_\pm ^\dagger (e_j)\right) \;. \end{aligned} \end{aligned}$$

A Bogoliubov transformation is then encoded by a $2 \times 2$ block matrix

21 $$\begin{aligned} \mathcal {V}= \begin{pmatrix} u & \quad v \\ \overline{v} & \quad \overline{u} \end{pmatrix} \;, \end{aligned}$$

with operators $u,v: \ell ^2 \rightarrow \ell ^2$. The case of unbounded *u*, *v* is treated later in Sect. 4. The Bogoliubov transformed operators are then given by

22 $$\begin{aligned} \begin{aligned} b_\pm ^\dagger (\varvec{f})&= A_\pm ^\dagger (\mathcal {V}(\varvec{f},0)) = a_\pm ^\dagger (u \varvec{f}) + a_\pm (v \overline{\varvec{f}}) \;,\\ b_\pm (\varvec{g})&= A_\pm (\mathcal {V}(\varvec{g},0)) =a_\pm ^\dagger (v \overline{\varvec{g}}) + a_\pm (u \varvec{g}) \;.\\ \end{aligned} \end{aligned}$$

In order for $\mathcal {V}$ to be a Bogoliubov transformation, we require that both $\mathcal {V}$ and $\mathcal {V}^*$ conserve the CAR/CCR, so

23 $$\begin{aligned} \left[ b_\pm (\varvec{f}),b_\pm ^\dagger (\varvec{g})\right] _\pm = \langle \varvec{f},\varvec{g}\rangle , \quad \left[ b_\pm (\varvec{f}),b_\pm (\varvec{g})\right] _\pm = 0 = \left[ b_\pm ^\dagger (\varvec{f}),b_\pm ^\dagger (\varvec{g})\right] _\pm , \end{aligned}$$

and the same, if in (22) $\mathcal {V}$ is replaced by $\mathcal {V}^*$. An explicit calculation shows that this conservation is equivalent to the Bogoliubov relations

24 $$\begin{aligned} \begin{aligned} u^*u \mp v^T \overline{v}&= 1 \;, \quad&u^*v \mp v^T \overline{u} = 0 \;,\\ u u^* \mp v v^*&= 1 \;, \quad&u v^T \mp v u^T =0 \;,\\ \end{aligned} \end{aligned}$$

with $(u^T)_{ij} = u_{ji}, \; (\overline{u})_{ij} = \overline{u_{ij}}, \; (u^*)_{ij} = \overline{u_{ji}}$ and the same for $v_{ij}$. Since $v^*v$ is self-adjoint, we have $v^*v = \overline{v^*v} = v^T \overline{v}$, so the first equation is equivalent to $u^*u \mp v^*v = 1$. The generalized creation and annihilation operators also allow for particularly easy “generalized CAR/CCR”: Using the standard scalar product on $\varvec{F},\varvec{G}\in \ell ^2 \oplus \ell ^2$:

25 $$\begin{aligned} \langle \varvec{F}, \varvec{G}\rangle = \left\langle \begin{pmatrix} \varvec{f}_1 \\ \varvec{f}_2 \end{pmatrix} , \begin{pmatrix} \varvec{g}_1 \\ \varvec{g}_2 \end{pmatrix} \right\rangle = \sum _j \left( \overline{f_{1,j}} g_{1,j} + \overline{f_{2,j}} g_{2,j}\right) , \end{aligned}$$

and $\mathcal {S}_- = {\textrm{id}}$, $$ \mathcal {S}_+ = \left( {\begin{smallmatrix} 1& \quad 0 \\ 0& \quad -1 \end{smallmatrix}} \right) $$, we obtain the generalized CAR/CCR:

26 $$\begin{aligned} \left[ A_\pm (\varvec{F}), A_\pm ^\dagger (\varvec{G})\right] _\pm = \langle \varvec{F}, \mathcal {S}_\pm \varvec{G}\rangle \;, \quad \left[ A_\pm (\varvec{F}), A_\pm (\varvec{G})\right] _\pm = \left[ A_\pm ^\dagger (\varvec{F}), A_\pm ^\dagger (\varvec{G})\right] _\pm = 0 \;. \end{aligned}$$

### Implementation on Fock Space

In the above notation, a Bogoliubov transformation is implementable (in the regular sense), if there exists a unitary operator $\mathbb {U}_\mathcal {V}: \mathscr {F}\rightarrow \mathscr {F}$, such that

27 $$\begin{aligned} \mathbb {U}_\mathcal {V}A^\dagger (\varvec{F}) \mathbb {U}_\mathcal {V}^* = A^\dagger (\mathcal {V}\varvec{F}) \;. \end{aligned}$$

We recap some of the basic steps of the implementation process presented in [45] for ${\textrm{tr}}(v^* v) < \infty$, as they have to be carried out in a slightly modified way for an implementation in the extended sense.

The main task within the implementation is to find the *Bogoliubov vacuum* $\Omega _\mathcal {V}\in \mathscr {F}$, which is the vector annihilated by all operators $b(\varvec{f})$:

28 $$\begin{aligned} b(\varvec{f}) \Omega _\mathcal {V}= \left( a^\dagger (u \varvec{f}) + a(v\overline{\varvec{f}})\right) \Omega _\mathcal {V}= 0 \quad \forall \varvec{f}\in \ell ^2. \end{aligned}$$

If we can find such an $\Omega _\mathcal {V}$, then it is an easy task to transform any product state vector $a^\dagger (\varvec{f}_1) \ldots a^\dagger (\varvec{f}_n) \Omega \in \mathscr {F}$ via:

29 $$\begin{aligned} \mathbb {U}_\mathcal {V}a^\dagger (\varvec{f}_1) \ldots a^\dagger (\varvec{f}_n) \Omega = b^\dagger (\varvec{f}_1) \ldots b^\dagger (\varvec{f}_n) \Omega _\mathcal {V}. \end{aligned}$$

The span of these state vectors (also called algebraic tensor product) is dense within $\mathscr {F}$, so we can transform any $\Psi \in \mathscr {F}$ by means of (29).

To find $\Omega _\mathcal {V}$, we decompose the transformation $\mathcal {V}$ into modes by constructing vectors $\varvec{f}_j$, such that $u \varvec{f}_j, v \overline{\varvec{f}_j}$ are proportional to the same normalized vector $\varvec{g}_j \in \ell ^2$, i.e.

30 $$\begin{aligned} \mathcal {V}\begin{pmatrix} \varvec{f}_j\\ 0 \end{pmatrix} = \begin{pmatrix} u \varvec{f}_j\\ \overline{v} \varvec{f}_j \end{pmatrix} = \begin{pmatrix} \mu _j \varvec{g}_j\\ \overline{\nu _j \varvec{g}_j} \end{pmatrix} \qquad \Leftrightarrow \qquad \mathbb {U}_\mathcal {V}\; a^\dagger (\varvec{f}_j) \; \mathbb {U}_\mathcal {V}^* = \mu _j a^\dagger (\varvec{g}_j) + \nu _j a(\varvec{g}_j) \;, \end{aligned}$$

where $\mu _j, \nu _j \in \mathbb {C}$. If (30) holds, we only have to solve $(\nu _j a^\dagger (\varvec{g}_j) + \mu _j a(\varvec{g}_j) )\Omega _\mathcal {V}$ for each $\varvec{g}_j$, separately.

#### Bosonic Case

Here, (30) can indeed be fulfilled: Following [45], introducing the complex conjugation $J \tilde{\varvec{h}}_j = J^* \tilde{\varvec{h}}_j = \overline{\tilde{\varvec{h}}_j}$, the operator $C:= u^* v J$ has an eigenbasis $(\varvec{f}_j)$ with $C \varvec{f}_j = \lambda _j \varvec{f}_j, \; \lambda _j \in \mathbb {C}$. One may further show that $\varvec{g}_j:= \mu _j^{-1} u \varvec{f}_j$ defines an orthonormal basis and that $v \overline{\varvec{f}_j} = v J \varvec{f}_f = \nu _j \varvec{g}_j$ where $\mu _j, \nu _j \geqslant 0$ are fixed by

31 $$\begin{aligned} \mu _j^2 - \nu _j^2 = 1 \;, \quad \lambda _j = \mu _j \nu _j \;. \end{aligned}$$

The condition $(\mu _j a(\varvec{g}_j) + \nu _j a^\dagger (\varvec{g}_j)) \Omega _\mathcal {V}= 0$ leads to one recursion relation per mode, which is formally solved by

32 $$\begin{aligned} \Omega _\mathcal {V}= \left( \prod _j \left( 1 - \tfrac{\nu _j^2}{\mu _j^2} \right) ^{1/4} \right) \exp {\left( -\sum _j \frac{\nu _j}{2 \mu _j} (a^\dagger (\varvec{g}_j))^2 \right) } \Omega \;. \end{aligned}$$

The Shale condition now indicates when $\Omega _\mathcal {V}$ lies in Fock space, which is if and only if $\sum _j \frac{\nu _j^2}{\mu _j^2}< \infty \Leftrightarrow \sum _j \nu _j^2 < \infty$. In that case, the transformation is implemented by [15, (3.1)]:

33 $$\begin{aligned} \mathbb {U}_\mathcal {V}= \exp { \left( - \sum _j \tfrac{\xi _j}{2} ((a^\dagger (\varvec{g}_j))^2 - (a(\varvec{g}_j))^2 ) \right) } \mathbb {U}_{\varvec{g}\varvec{f}} =: \prod _{j \in \mathbb {N}} \mathbb {U}_{j,\mathcal {V}} \;, \end{aligned}$$

34 $$\begin{aligned} {\text {with}} \qquad \sinh \xi _j := \nu _j \quad \Rightarrow \quad \cosh \xi _j := \mu _j \;, \end{aligned}$$

and where $\mathbb {U}_{\varvec{g}\varvec{f}}: \mathscr {F}\rightarrow \mathscr {F}$ is the unitary basis change transformation

35 $$\begin{aligned} \mathbb {U}_{\varvec{g}\varvec{f}}: \varvec{f}_{j_1} \otimes \cdots \otimes \varvec{f}_{j_n} \mapsto \varvec{g}_{j_1} \otimes \cdots \otimes \varvec{g}_{j_n} \quad \forall \; j_1, \ldots , j_n \in \mathbb {N}. \end{aligned}$$

For a more general discussion on $\mathbb {U}_\mathcal {V}$, we refer the reader to [10, Theorem 16.47].

#### Fermionic Case

In the fermionic case, following [45], we can find a common orthonormal eigenbasis $(\varvec{f}_j)_{j \in J}$ of $C^*C$ (eigenvalues $\lambda _j^2$), of $u^*u$ (eigenvalues $\mu _j^2$), and of $v^*v$ (eigenvalues $\nu _j^2 = 1 - \mu _j^2$). The index set can be split as $J = J' \cap J'' \subseteq \mathbb {N}$ with $J''$ containing all indices of zero eigenvectors and $J'$ those of nonzero eigenvectors. Further, the $j \in J'$ can be rearranged in pairs as $J':= \{j \; \mid \; j=2i \vee j=2i-1, \; i \in I'\}$ such that

36 $$\begin{aligned} C \varvec{f}_{2i} = \lambda _{2i} \varvec{f}_{2i-1} \;, \quad C \varvec{f}_{2i-1} = - \lambda _{2i} \varvec{f}_{2i}, \end{aligned}$$

for some $I' \subseteq \mathbb {N}$. For $j \in J''$, two cases may occur: If $\nu _j = 1$, then we have a particle–hole transformation, for which we write $j \in J''_1$ and get

37 $$\begin{aligned} \mathcal {V}\begin{pmatrix} \varvec{f}_j \\ 0 \end{pmatrix} = \begin{pmatrix} 0 \\ \overline{\varvec{\eta }_j} \end{pmatrix} \;, \quad j \in J''_1 \;, \end{aligned}$$

for a suitable choice of the phase $\varvec{\eta }_j = e^{i \varphi } v \overline{\varvec{f}_j}$. The case $\nu _j = 0$ will be denoted by $j \in J''_0 = J'' {\setminus } J''_1$, and we have

38 $$\begin{aligned} \mathcal {V}\begin{pmatrix} \varvec{f}_j \\ 0\end{pmatrix} = \begin{pmatrix} \varvec{\eta }_j \\ 0 \end{pmatrix}, \quad j \in J''_0, \end{aligned}$$

for a suitable phase choice of $\varvec{\eta }_j = e^{i \varphi } u \varvec{f}_j$. In case $j \in J'$ with $i \in I' \Rightarrow \lambda _{2i} \ne 0, \mu _{2i} \ne 0$ (Cooper pair), we may define the normalized vectors

39 $$\begin{aligned} \varvec{\eta }_{2i} := \alpha _i^{-1} \; u \varvec{f}_{2i} \;, \qquad \varvec{\eta }_{2i-1} := \alpha _i^{-1} \; u \varvec{f}_{2i-1} \;, \end{aligned}$$

where $\alpha _i, \beta _i > 0$, $\alpha _i = \mu _{2i} = \mu _{2i-1}$, $\alpha _i^2 + \beta _i^2 = 1$, and for which

40 $$\begin{aligned} \mathcal {V}\begin{pmatrix} \varvec{f}_{2i} \\ 0\end{pmatrix} = \begin{pmatrix} \alpha _i \; \varvec{\eta }_{2i} \\ \overline{\beta _i \; \varvec{\eta }_{2i-1}} \end{pmatrix} \;, \qquad \mathcal {V}\begin{pmatrix} \varvec{f}_{2i-1} \\ 0\end{pmatrix} = \begin{pmatrix} \alpha _i \; \varvec{\eta }_{2i-1} \\ \overline{-\beta _i \; \varvec{\eta }_{2i}} \end{pmatrix} \;, \qquad i \in I' \;. \end{aligned}$$

Relations (37), (38) and (40) now replace (30). The Bogoliubov vacuum is

41 $$\begin{aligned} \Omega _\mathcal {V}= \left( \prod _{j \in J''_1} a^\dagger (\varvec{\eta }_j) \right) \left( \prod _{i \in I'} \left( \alpha _i - \beta _i a^\dagger (\varvec{\eta }_{2i}) a^\dagger (\varvec{\eta }_{2i-1})\right) \right) \Omega \;, \end{aligned}$$

and the implementer is

42 $$\begin{aligned} \begin{aligned} \mathbb {U}_\mathcal {V}&= \left( \prod _{j \in J''_1} \left( a^\dagger (\varvec{\eta }_j) + a(\varvec{\eta }_j)\right) \right) \exp \left( -\sum _{i \in I'} \xi _i \left( a^\dagger (\varvec{\eta }_{2i}) a^\dagger (\varvec{\eta }_{2i-1}) - a(\varvec{\eta }_{2i-1}) a(\varvec{\eta }_{2i})\right) \right) \mathbb {U}_{\varvec{\eta }\varvec{f}}\\ \mathbb {U}_\mathcal {V}&=: \left( \prod _{j \in J''} \mathbb {U}_{j,\mathcal {V}}\right) \left( \prod _{i \in I'} \mathbb {U}_{2i,2i-1,\mathcal {V}} \right) \;, \quad {\text {with}} \quad \sin \xi _i := \beta _i \quad \Rightarrow \quad \cos \xi _i := \alpha _i \;, \end{aligned} \end{aligned}$$

and where $\mathbb {U}_{\varvec{\eta }\varvec{f}}$ is the unitary basis change transformation

43 $$\begin{aligned} \mathbb {U}_{\varvec{\eta }\varvec{f}}: \varvec{f}_{j_1} \otimes \cdots \otimes \varvec{f}_{j_n} \mapsto \varvec{\eta }_{j_1} \otimes \cdots \otimes \varvec{\eta }_{j_n} \quad \forall \; j_1, \ldots , j_n \in J. \end{aligned}$$

## Bogoliubov Transformations: Extended

In the extended case, *v* is possibly unbounded, so we must show that the Bogoliubov relations (24) survive the extension. We do this in Sect. 4.1, while preparing the spectral decomposition for the final ITP construction. In Sect. 4.2, we define an extended ^*^ -algebra $\overline{\mathcal {A}}_{\varvec{e}}$ of creation and annihilation operator products, and in Sect. 4.3, we finish the construction of $\widehat{\mathscr {H}}$, with respect to a given Bogoliubov transformation $\mathcal {V}$.

### Extension of the Bogoliubov Relations

**Fig. 2:**
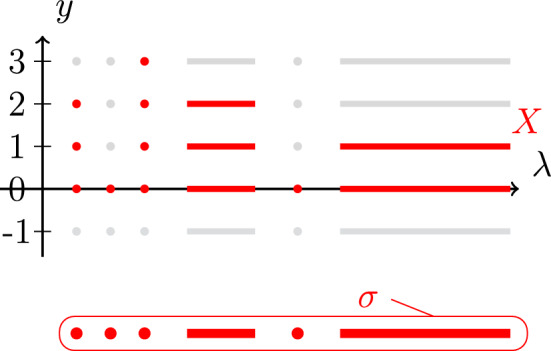
The spectral set *X* for a generic spectrum of |*C*|

Throughout the following construction, we will assume that $v^*v$ is densely defined and self-adjoint. In that case, we can define the self-adjoint operators $C^*C:= v^*v (1 \pm v^*v)$ and $|C| = \sqrt{C^*C}$ by spectral calculus. By the spectral theorem in the form of [47, Thm. 10.9], we may then decompose $\ell ^2$ as a direct integral

44 $$\begin{aligned} \ell ^2 = \int ^\oplus _{\sigma (|C|)} \mathbb {C}^n \; {\textrm{d}}\mu _1(\lambda ) \;, \end{aligned}$$

where $\sigma (|C|) = \sigma$ is the spectrum of |*C*| , $\mu _1$ is a suitable measure on it and $n: \sigma \rightarrow \mathbb {N}\cup \{\infty \}$ [47, Def. 7.18] is a measurable dimension function. Put differently, as visualized in Fig. 2, we can find a spectral set $X = \bigcup _{\lambda \in \sigma } \{\lambda \} \times Y_\lambda \subset \mathbb {R}^2$ with $Y_\lambda \subseteq \mathbb {Z}, |Y_\lambda | = n(\lambda )$ accounting for multiplicity and unitary maps^3^

45 $$\begin{aligned} U_{X \varvec{f}}: \ell ^2 \rightarrow L^2(X) \;, \quad U_{\varvec{f}X} = U_{X \varvec{f}}^{-1} \;, \end{aligned}$$

such that

46 $$\begin{aligned} |C| = U_{\varvec{f}X} \lambda U_{X \varvec{f}} \;, \end{aligned}$$

with $\lambda$ being the operator on $L^2(X)$ that multiplies by $\lambda (x)$. In addition, we denote $Y = \bigcup _{\lambda \in \sigma } Y_\lambda \subseteq \mathbb {Z}$ with |*Y*| being an upper bound for the multiplicity of any eigenvalue. Note that the $\lambda$ here correspond to the $\lambda _j$ in Sect. 3.2.

We also make use of the formulation [47, Thm. 10.4] of the spectral theorem, which provides us with a projection-valued measure $P_{|C|}$, such that

47 $$\begin{aligned} |C| = \int _X \lambda (x) \; dP_{|C|}(x) = \int _{\sigma \times Y} \lambda \; {\textrm{d}}P_{|C|}(\lambda , y) \;. \end{aligned}$$

Here, we choose $Y \subseteq \mathbb {Z}$, so $X \subset \mathbb {R}^2$ consists of “lines” with distance 1.

The case $\lambda = 0$ will turn out to be critical, whence we define

48 $$\begin{aligned} X_{{\textrm{crit}}} := \{ x = (\lambda ,y) \in X \; \mid \; \lambda = 0\} \;, \quad X_{{\textrm{reg}}} := X \setminus X_{{\textrm{crit}}} \;. \end{aligned}$$

Our (dense) space of test functions on the spectral set is given by:

49 $$\begin{aligned} \mathcal {D}_X := C_c^\infty (X_{{\textrm{crit}}}) \otimes C_c^\infty (X_{{\textrm{reg}}}) \;. \end{aligned}$$

The corresponding test function space in $\ell ^2$ is

50 $$\begin{aligned} \mathcal {D}_{|C|} := U_{\varvec{f}X} \mathcal {D}_X. \end{aligned}$$

For non-open *X*, we interpret (49) in the same way as the definition of $\mathcal {E}(X)$ (131):

$$\begin{aligned} C_c^\infty (X) := C_c^\infty (\mathbb {R}^2) / \{\phi \; \mid \; \phi (x) = 0 \; \forall x \in X \} \;. \end{aligned}$$

#### Lemma 4.1

(Bogoliubov relations (24) survive the extension) Suppose, *u* and *v* are defined on a common dense domain $\mathcal {D}\subseteq \ell ^2$, such that $v^*v$ is densely defined and self-adjoint, and such that the linear operator

51 $$\begin{aligned} \mathcal {V}= \begin{pmatrix} u & \quad v \\ \overline{v} & \quad \overline{u} \end{pmatrix} \;, \quad \mathcal {V}: \mathcal {D}\oplus \mathcal {D}\rightarrow \ell ^2 \oplus \ell ^2, \end{aligned}$$

defines a Bogoliubov transformation. That means, both $\mathcal {V}$ and $$ \mathcal {V}^* = \left( {\begin{smallmatrix} u^* &  v^T \\ v^* &  u^T \end{smallmatrix}} \right) $$ preserve the CAR/CCR, see (20), (23).

Then $u, v, \overline{u}, \overline{v}, u^*, v^*, u^T$ and $v^T$ are well-defined on all of $\mathcal {D}_{|C|}$, constructed above (50). Further, the Bogoliubov relations (24) hold as a weak operator identity on $\mathcal {D}_{|C|}$. Conversely, the extended Bogoliubov relations imply conservation of the CAR/CCR under both $\mathcal {V}$ and $\mathcal {V}^*$.

#### Proof

Well-definedness of *u*, *v* on $\mathcal {D}_{|C|}$ follows from the polar decompositions

52 $$\begin{aligned} v = U_v |v| \;, \quad u = U_u |u|, \end{aligned}$$

with unitary $U_v, U_u: L^2(X) \rightarrow \ell ^2$. The operators $|v| = \sqrt{v^*v}$ and $|u| = \sqrt{u^*u} = \sqrt{1 \pm v^*v}$ on $L^2(X)$ are bounded in the fermionic case ($-$), so *u* and *v* are defined on all of $\ell ^2$. In the bosonic case ($+$), they are spectral multiplications by

53 $$\begin{aligned} \nu (\lambda ) = \sqrt{-\frac{1}{2} + \sqrt{\frac{1}{4} + \lambda ^2}} \quad {\text {and}} \quad \mu (\lambda ) = \sqrt{\frac{1}{2} + \sqrt{\frac{1}{4} + \lambda ^2}} \;, \end{aligned}$$

which are bounded on each bounded interval in $\lambda$. Therefore, |*v*| and |*u*| map $\mathcal {D}_X$ into itself, and by definition (50) of $\mathcal {D}_{|C|}$, the operators *v* and *u* map $\mathcal {D}_{|C|} \rightarrow \ell ^2$. Well-definedness of $\overline{u}$ and $\overline{v}$ on $\mathcal {D}_{|C|}$ follows analogously and the domains of the adjoints $u^*, v^*, u^T$ and $v^T$ contain the domain of the respective original operators, so they all contain $\mathcal {D}_{|C|}$. The CAR/CCR conservation then follows by a direct computation. Checking that (24) indeed holds as weak operator identities on $\mathcal {D}_{|C|}$ is straightforward to check. $\square$

**Fig. 3:**
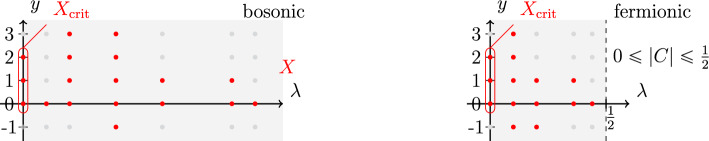
Left: Discrete spectrum of |*C*| in the bosonic case. Right: In the fermionic case, $|C| \leqslant \tfrac{1}{2}$ holds

#### Lemma 4.2

Let $v^*v$ be self-adjoint. Then $C = u^* v J$ and $C^*C$ are well-defined operators on $\mathcal {D}_{|C|}$, where *J* denotes complex conjugation. The spectrum of *C* is contained in the real axis for bosons and the imaginary axis for fermions.

Further, if $v^*v$ has countable spectrum, then also $C, C^*C$ and |*C*| have countable spectrum, see Fig. 3.

#### Proof

By the Bogoliubov relations, $C^*C = v^*v \pm (v^*v)^2$ holds, wherever it is defined. Now, $\lambda \mapsto \lambda \pm \lambda ^2$ is smooth apart from the critical points 0 (bosons) or 0 and 1 (fermions). The condition $\varvec{\phi }\in \mathcal {D}_{|C|}$ means that the corresponding spectral function $\phi _X$ has compact support and is smooth apart from the critical points. This property is preserved by an application of $C^*C$, so $C^*C: \mathcal {D}_{|C|} \rightarrow \mathcal {D}_{|C|}$ is well-defined. Hence, also $|C| = \sqrt{C^*C}$ is well-defined, and by a polar decomposition also $C = U_C |C|$ with $U_C: \ell ^2 \rightarrow \ell ^2$ unitary.

In the bosonic case, *C* is symmetric by the Bogoliubov relations, so $C^*C = C^2$ and further, $\sigma (C)$ is a subset of the preimage of $\sigma (C^2) \subseteq [0,\infty )$ under the complex map $z \mapsto z^2$. This preimage is contained within the real axis.

In the fermionic case, the Bogoliubov relations imply $C^* = -C$, so $C^*C = -C^2$. That means, $\sigma (C)$ lies within the preimage of $\sigma (C^2) \subseteq [0,\infty )$ under the map $z \mapsto -z^2$, which is contained within the imaginary axis.

Now, suppose $\sigma (v^*v)$ is countable. Then, $\sigma (|C|)$ is the image of $\sigma (v^*v)$ under the map $z \mapsto \sqrt{z(1 \pm z)}$, which sends at most 2 arguments to the same value, so $\sigma (|C|)$ is also countable. $\square$

#### Remark 4.3

For fermions, $u^*u + v^*v = 1$ implies that $v^*v$ is bounded, so $v^*v$ and also $u^*u$ can be defined on all of $\ell ^2$. Hence, also *v* and *u* are defined on all of $\ell ^2$, so the Bogoliubov relations (24) hold as a strong operator identity on $\ell ^2$.

#### Remark 4.4

For bosons, it is not obvious that the Bogoliubov relations (24) hold as a strong operator identity on a dense domain of $\ell ^2$. In fact, it does not always hold as a strong operator identity on $\mathcal {D}_{|C|}$: As a counter-example, let $v e_j:= j e_j$, with respect to the canonical basis $(e_j)_{j \in \mathbb {N}}$ and let $u = U_u |u|$ such that $U_u^* e_1 = c \; \sum _j j^{-1/2 - \varepsilon } e_j$ for some $\varepsilon > 0$. A direct calculation then shows

54 $$\begin{aligned} u^*v e_1 = |u| U_u^* e_1 = \sum _j \sqrt{1 + j^2} j^{-1/2 - \varepsilon } e_j \;. \end{aligned}$$

For $\varepsilon \leqslant 1$, this is obviously not in $\ell ^2$, so $u^*v$ is ill-defined on $e_1 \in \mathcal {D}_{|C|}$.

### Extension of the Operator Algebra

In Sect. 2.1, we defined a ^*^ -algebra (bosonic) or $C^*$-algebra (fermionic) $\mathcal {A}$ generated by $a^\dagger _\pm (f), a_\pm (f), f \in \mathfrak {h}$. On our ITP spaces, we will encounter formal expressions in creation and annihilation operators that belong to a larger algebra $\overline{\mathcal {A}}_{\varvec{e}}$, which is defined with respect to a basis $\varvec{e}= (\varvec{e}_j)_{j \in \mathbb {N}} \subset \ell ^2$. We introduce the shorthand notations $a_j:= a(\varvec{e}_j), a^\dagger _j:= a^\dagger (\varvec{e}_j)$, and consider the set of finite operator products

55 $$\begin{aligned} \Pi _{\varvec{e}} := \left\{ a_{j_1}^\sharp \ldots a_{j_m}^\sharp \; \mid \; j_{\ell } \in \mathbb {N}\right\} . \end{aligned}$$

Then $\overline{\mathcal {A}}_{\varvec{e}}$ is defined as the set of all complex-valued maps

56 $$\begin{aligned} \overline{\mathcal {A}}_{\varvec{e}} := \{ H: \Pi _{\varvec{e}} \rightarrow \mathbb {C}\} \;. \end{aligned}$$

We formally write its elements as infinite sums

57 $$\begin{aligned} H = \sum _{m \in \mathbb {N}} \sum _{j_1,\ldots , j_m \in \mathbb {N}} H_{j_1,\ldots , j_m} a^\sharp _{j_1} \ldots a^\sharp _{j_m} \;. \end{aligned}$$

$\overline{\mathcal {A}}_{\varvec{e}}$ is made a ^*^ -algebra by the involution58It is easy to see that $\overline{\mathcal {A}}_{\varvec{e}}$ extends $\mathcal {A}$. Resolving each $a^\sharp (f_j)$ with respect to the basis $\varvec{e}$, we obtain a countable sum of the form (57), that contains each term $a^\sharp _{j_1} \ldots a^\sharp _{j_m}$ at most once.

Of particular interest will be elements of $\overline{\mathcal {A}}_{\varvec{e}}$ corresponding to finite sums. For $a^\dagger (\varvec{\phi }) = \sum _{j \in \mathbb {N}} \phi _j a^\dagger _j, \varvec{\phi }\in \ell ^2$, this sum is finite if and only if

59 $$\begin{aligned} \varvec{\phi }\in \mathcal {D}_{\varvec{e}} := \left\{ \varvec{\phi }\in \ell ^2 \; \mid \; \phi _j = 0 \; {\text {for all but finitely many}} \; j \in \mathbb {N}\right\} . \end{aligned}$$

Here, the index $\varvec{e}$ in $\mathcal {D}_{\varvec{e}}$ emphasizes that we are working with respect to the basis $(\varvec{e}_j)_{j \in \mathbb {N}}$. If $(\varvec{e}_j)_{j \in \mathbb {N}}$ is an orthonormal eigenbasis of |*C*| , then $\mathcal {D}_{\varvec{e}} = \mathcal {D}_{|C|}$, since both domains are spanned by finite linear combinations of eigenvectors of $C^*C$.

### Final ITP Construction and Operator Lift

Next, we fix the final definitions for our ITP spaces $\widehat{\mathscr {H}}$ and define products of $a^\dagger (\varvec{\phi }), a(\varvec{\phi })$ with $\varvec{\phi }\in \mathcal {D}_{\varvec{e}}$ on suitable subspaces of them. Within these definitions, **we assume that** $v^*v$ **has countable spectrum**, so Lemma 4.2 applies and |*C*| has countable spectrum.

As argued around (47), $X \subseteq \sigma \times Y$ is countable and there exists an orthonormal eigenbasis $(\varvec{f}_j)_{j \in \mathbb {N}}$.

For bosons we follow the construction of [45], replacing the argument “*C* is Hilbert–Schmidt” by “ |*C*| has countable spectrum”, which renders an orthonormal basis $\varvec{g}= (\varvec{g}_j)_{j \in \mathbb {N}}$ as in Sect. 3. The construction uses that by Lemma 4.1, the Bogoliubov relations still hold as a weak operator identity. Now, $\varvec{g}$ takes the role of $\varvec{e}$ in $\mathcal {D}_{\varvec{e}}$ and $\overline{\mathcal {A}}_{\varvec{e}}$.

#### Definition 4.5

The **bosonic infinite tensor product space** is given by

60 $$\begin{aligned} \widehat{\mathscr {H}} = \prod _{k \in \mathbb {N}}^\otimes \mathscr {H}_k = \prod _{k \in \mathbb {N}}^\otimes \mathscr {F}(\{\varvec{g}_k\}), \end{aligned}$$

Note that the sequence $(e_{k,n})_{n \in \mathbb {N}_0}$ of *n*-particle basis vectors is a canonical basis of each one-mode Fock space $\mathscr {H}_k$, and can be used to describe elements of $\widehat{\mathscr {H}}$.

For fermions the construction of [45], with ”$C^*C$ is trace class” replaced by “ |*C*| has countable spectrum” (which is true by Lemma 4.2), yields an orthonormal basis $(\varvec{\eta }_j)_{j \in J}$ with countable $J \subseteq \mathbb {N}$ as in Sect. 3.2.2. Here, $\varvec{\eta }$ takes the role of $\varvec{e}$ in $\mathcal {D}_{\varvec{e}}$ and $\overline{\mathcal {A}}_{\varvec{e}}$.

The ITP construction is then a bit more delicate, since for each Cooper pair $i \in I'$ (so $j \in J'$), we must introduce a separate Fock space $\mathscr {F}(\{\varvec{\eta }_{2i-1}\}) \otimes \mathscr {F}(\{\varvec{\eta }_{2i}\}) \cong \mathbb {C}^4$ (see Remark 5.8). We index all $j \in J''$ and $i \in I'$ by a corresponding *k*(*i*) or *k*(*j*) , such that all $k \in \mathbb {N}$ are used and take the tensor product over those *k*:

#### Definition 4.6

The **fermionic infinite tensor product space** is given by

61 $$\begin{aligned} \widehat{\mathscr {H}} = \prod _{k \in \mathbb {N}}^\otimes \mathscr {H}_k = \left( \prod _{j \in J''}^\otimes \mathscr {F}(\{\varvec{\eta }_j\}) \right) \otimes \left( \prod _{i \in I'}^\otimes \mathscr {F}(\{\varvec{\eta }_{2i-1}\}) \otimes \mathscr {F}(\{\varvec{\eta }_{2i}\}) \right) . \end{aligned}$$

For a one-mode Fock space $\mathscr {H}_{k(j)}:= \mathscr {F}(\{\varvec{\eta }_j\})$, the pair $(e_{k,0}, e_{k,1})$ forms a basis of each $\mathscr {H}_k$, while for a two-mode Fock space $\mathscr {H}_{k(i)}:= \mathscr {F}(\{\varvec{\eta }_{2i-1}\}) \otimes \mathscr {F}(\{\varvec{\eta }_{2i}\})$, such a basis is given by $(e_{k,0,0}, e_{k,1,0}, e_{k,0,1}, e_{k,1,1})$, with the two indices denoting the particle numbers per mode.

Our next challenge is to lift the one-mode creation and annihilation operators $a_j^\dagger , a_j$ defined on the one- or two-mode Fock space $\mathscr {H}_k$ to $\widehat{\mathscr {H}}$.

#### Lemma 4.7

Consider a (possibly unbounded) operator $A_{j,j}: \mathscr {H}_j \supset {\textrm{dom}}(A_{j,j}) \rightarrow \mathscr {H}_j$. Then, for $\Psi ^{(m)}_j \in {\textrm{dom}}(A_{j,j})$,

62 $$\begin{aligned} A_j \Psi ^{(m)} := \Psi ^{(m)}_1 \otimes \cdots \otimes \Psi ^{(m)}_{j-1} \otimes A_{j,j} \Psi ^{(m)}_j \otimes \Psi ^{(m)}_{j+1} \otimes \cdots , \end{aligned}$$

is independent of the choice of a *C*-sequence $(\Psi ^{(m)}) = (\Psi ^{(m)}_k)_{k \in \mathbb {N}}$ representing $\Psi ^{(m)}$, and defines an operator $A_j$ by linearity on

63 $$\begin{aligned} \Psi \in {\textrm{dom}}(A_j) := \Big \{ \Psi = \sum _{m \in \mathcal {M}} d_m \Psi ^{(m)} \in \widehat{\mathscr {H}} \; \Big \vert \; \Big \Vert \sum _{m \in \mathcal {M}} d_m A_j \Psi ^{(m)} \Big \Vert < \infty \Big \} \;, \end{aligned}$$

where $\mathcal {M}\subseteq \mathbb {N}$, $d_m \in \mathbb {C}$ and $\Psi ^{(m)}$ being such that $A_j \Psi ^{(m)}$ is well-defined by (62).

#### Proof

For a fixed choice of $(\Psi ^{(m)})$ representing $\Psi ^{(m)}$ such that $\Psi ^{(m)}_j \in {\textrm{dom}}(A_{j,j})$, well-definedness of $A_j \Psi ^{(m)}$ is easy to see. By Lemma A.2, we can now represent $\Psi = \sum _{m \in \mathcal {M}} d_m \Psi ^{(m)}$. And if $\sum _{m \in \mathcal {M}} d_m A_j \Psi ^{(m)}$ converges, then it is independent of the representation, since $A_j$ is linear. So ${\textrm{dom}}(A_j)$ and $A_j \Psi$ are well-defined.

It remains to prove that $A_k \Psi ^{(m)}$ (and hence $A_k \Psi$) is independent of the choice of $(\Psi ^{(m)})$ representing $\Psi ^{(m)}$. For $m \in \mathcal {M}$, consider a second representative *C*-sequence $(\tilde{\Psi }^{(m)})$ with $\tilde{\Psi }^{(m)} = \Psi ^{(m)}$. By Proposition A.1, $\tilde{\Psi }_k^{(m)} = c_k \Psi _k^{(m)}$ for some $c_k \in \mathbb {C}$ with $\prod _{k \in \mathbb {N}} c_k = 1$. By linearity, $A_{j,j} \tilde{\Psi }_k^{(m)} = c_k A_{j,j} \Psi _k^{(m)}$, so also $A_j \Psi ^{(m)}$ and $A_j \tilde{\Psi }^{(m)}$ defined by (62) just differ by the sequence of complex factors $(c_k)_{k \in \mathbb {N}}$ with $\prod _{k \in \mathbb {N}} c_k = 1$. Hence, according to Proposition A.1, they correspond to the same functional $A_j \tilde{\Psi }^{(m)} = A_j \Psi ^{(m)}$. $\square$

The operators $a_j, a^\dagger _j$ may be unbounded. We thus need to carefully choose a domain to make them bounded, by restricting the space of allowed $\Psi$.

#### Definition 4.8

In the bosonic case, the **space** $\mathcal {S}^\otimes$ **with rapid decay in the particle number** is defined as

64 $$\begin{aligned} \mathcal {S}^\otimes := \left\{ \Psi \in \bigcap _{\begin{array}{c} n \in \mathbb {N}\\ k \in \mathbb {N}_0 \end{array}} {\textrm{dom}}(N_k^n) \subseteq \widehat{\mathscr {H}} \; \Big \vert \; \Vert N_k^n \Psi \Vert \leqslant c_{k,n} \Vert \Psi \Vert \; \forall k \in \mathbb {N}, \; n \in \mathbb {N}_0 \right\} , \end{aligned}$$

where $c_{k,n} > 0$ are suitable constants for each *n* and *k*, and $N_k$ is the number operator on $\mathscr {H}_k$, lifted to $\widehat{\mathscr {H}}$. The lift is possible by Lemma 4.7, which also allows defining $N_k^n$.

In the fermionic case, the maximum particle number per mode is 1, so we always have rapid decay and simply set

65 $$\begin{aligned} \mathcal {S}^\otimes := \widehat{\mathscr {H}}. \end{aligned}$$

#### Lemma 4.9

(Products of $a^\dagger , a$ are well-defined on the ITP space) Consider the ITP space $\widehat{\mathscr {H}} \supseteq \mathcal {S}^\otimes$ corresponding to the basis $(\varvec{e}_j)_{j \in \mathbb {N}}$, which is $(\varvec{g}_j)_{j \in \mathbb {N}}$ (bosonic) or $(\varvec{\eta }_j)_{j \in \mathbb {N}}$ (fermionic). Then we can lift $a_j$ and $a_j^\dagger$ to $\widehat{\mathscr {H}}$ and for $\varvec{\phi }\in \mathcal {D}_{\varvec{g}}$ or $\mathcal {D}_{\varvec{\eta }}$ (defined in (59)), the expressions

66 $$\begin{aligned} a^\dagger (\varvec{\phi }) = \sum _j \phi _j a^\dagger _j \;, \qquad a(\varvec{\phi }) = \sum _j \overline{\phi _j} a_j \;, \end{aligned}$$

define linear operators $a^\dagger (\varvec{\phi }): \mathcal {S}^\otimes \rightarrow \mathcal {S}^\otimes$ and $a(\varvec{\phi }): \mathcal {S}^\otimes \rightarrow \mathcal {S}^\otimes$.

#### Proof

First, note that the sum over *j* in (66) is finite by definition of $\mathcal {D}_{\varvec{g}}$ and $\mathcal {D}_{\varvec{\eta }}$.

In the fermionic case, $a_j, a_j^\dagger$ are bounded. So by [3, Lemma 5.1.1], we can lift them to bounded operators on $\mathcal {S}^\otimes = \widehat{\mathscr {H}}$, and also the finite linear combination in (66) is a bounded operator on $\mathcal {S}^\otimes$.

In the bosonic case, where $j = k$, we have

67 $$\begin{aligned} \Vert a^\dagger _k \Psi \Vert = \Vert \sqrt{N_k+1} \Psi \Vert \leqslant \Vert (N_k+1) \Psi \Vert \leqslant (c_{k,1} + 1) \Vert \Psi \Vert \;, \end{aligned}$$

so

68 $$\begin{aligned} \Vert a^\dagger (\varvec{\phi }) \Psi \Vert \leqslant \left( \sum _{k: \phi _k \ne 0} |\phi _k| (c_{k,1} + 1) \right) \Vert \Psi \Vert =: c_1 \Vert \Psi \Vert \;, \end{aligned}$$

where the sum over *k* contains only finitely many nonzero terms, so we may call it $c_1 > 0$. Hence, $a^\dagger (\varvec{\phi }) \Psi \in \widehat{\mathscr {H}}$ is well-defined. It remains to establish rapid decay. Now,

69 $$\begin{aligned} \begin{aligned} \Vert N_k^n a^\dagger _k \Psi \Vert&= \Vert N_k^n \sqrt{N_k+1} \Psi \Vert \leqslant \Vert N_k^{n+1} \Psi \Vert + \Vert N_k^n \Psi \Vert \leqslant (c_{k,n+1} + c_{k,n}) \Vert \Psi \Vert \\&\leqslant (c_{k,n+1} + c_{k,n}) \Vert a^\dagger _k \Psi \Vert \;, \end{aligned} \end{aligned}$$

so by summing over *k*, the rapid decay condition is again satisfied and $a^\dagger (\varvec{\phi }) \Psi \in \mathcal {S}^\otimes$.

For $a(\varvec{\phi }) \Psi \in \mathcal {S}^\otimes$, the same finite-sum argument can be used to obtain $a(\varvec{\phi }) \Psi \in \widehat{\mathscr {H}}$. However, verifying rapid decay needs a bit more attention, since $\Vert a^\dagger _k \Psi \Vert \leqslant \Vert \Psi \Vert$ in (69) does not generalize to $a_k$, see also Remark 4.10. However, for all *k* with $a_k \Psi \ne 0$ and $\phi _k \ne 0$, there is a fixed ratio $\frac{\Vert \Psi \Vert }{\Vert a_k \Psi \Vert } =: d_k > 0$. So with $d:= \max _k d_k$,

70 $$\begin{aligned} \begin{aligned} \Vert N_k^n a_k \Psi \Vert&\leqslant (c_{k,n+1} + c_{k,n}) \Vert \Psi \Vert \leqslant d \cdot (c_{k,n+1} + c_{k,n}) \Vert a_k \Psi \Vert \;. \end{aligned} \end{aligned}$$

For $a_k \Psi = 0$, the inequality is trivial. A finite sum over *k* then establishes $a(\varvec{\phi }) \Psi \in \mathcal {S}^\otimes$. $\square$

#### Remark 4.10

The condition $\varvec{\phi }\in \mathcal {D}_{\varvec{f}}$ is indeed necessary, meaning we may not just allow any $\varvec{\phi }\in \ell ^2$ inside $a^\sharp (\varvec{\phi })$, as the following counterexample shows: For the bosonic case ($j = k$), consider $\phi _k = \frac{1}{k}$, so $\varvec{\phi }\in \ell ^2 {\setminus } \mathcal {D}_{\varvec{f}}$. For each mode *k*, consider the coherent state $\Psi _k$ defined sector-wise by

71 $$\begin{aligned} \Psi _k^{(N_k)} = e^{-\frac{\alpha _k}{2}} (N_k!)^{-\frac{1}{2}} \alpha _k^{\frac{N_k}{2}} \;, \end{aligned}$$

where all $\alpha _k \in \mathbb {R}$ are set equal to the same $\alpha _k = \alpha > 0$ and where $\Vert \Psi _k \Vert _k = 1$. Then, define the ITP $\Psi = \prod ^\otimes _{k \in \mathbb {N}} \Psi _k$. It is easy to see that $\Psi$ satisfies the rapid decay condition (64), as for each $\Psi _k$, $\Vert \Psi _k^{(N_k)} \Vert _k$ decays exponentially in $N_k$. But still, $(\alpha _k)_{k \in \mathbb {N}} \notin \ell ^2$, so we may think of $\Psi$ as a “coherent state with a large displacement”, living outside the Fock space. It is a well-known fact about coherent states that $a_k \Psi = \alpha \Psi$, so

72 $$\begin{aligned} \Vert a(\varvec{\phi }) \Psi \Vert = \Big \Vert \sum _k \overline{\phi _k} \alpha \Psi \Big \Vert = \alpha \sum _k \frac{1}{k} \Vert \Psi \Vert = \infty \;. \end{aligned}$$

Hence, $a(\varvec{\phi })$ is ill-defined on $\Psi$.

The same happens with any coherent state product (71) and any $\varvec{\phi }$, where $\sum _k \overline{\phi _k} \alpha _k = \infty$. In particular, the space of allowed $(\phi _k)_{k \in \mathbb {N}}$ is dual to the one of allowed $(\alpha _k)_{k \in \mathbb {N}}$.

#### Remark 4.11

It is also possible to define $a^\sharp (\varvec{\phi })$ for more general $\varvec{\phi }$, if one restricts the space $\mathcal {S}^\otimes$. For instance, it is not too difficult to see that imposing uniform rapid decay via

73 $$\begin{aligned} \Psi \in \mathcal {S}^\otimes _{{\textrm{uni}}} := \left\{ \Psi \in \widehat{\mathscr {H}} \; \Bigg \vert \; \Vert N_k^n \Psi \Vert \leqslant c_n \Vert \Psi \Vert \; \forall n, k \in \mathbb {N}\right\} \end{aligned}$$

yields a well-defined product $a^\sharp (\varvec{\phi }_1) \ldots a^\sharp (\varvec{\phi }_n) \Psi \in \widehat{\mathscr {H}}$ for any $\varvec{\phi }_1, \ldots , \varvec{\phi }_n \in \ell ^1$.

Alternatively, one may allow for $\varvec{\phi }_1, \ldots , \varvec{\phi }_n \in \ell ^p$, $p \in (1,2]$ by restricting to

74 $$\begin{aligned} \Psi \in \mathcal {S}^\otimes _q := \Big \{ \Psi \in \widehat{\mathscr {H}} \; \Big \vert \; \Vert (N_k+1)^{n/2} \Psi \Vert \leqslant c_k^n \Vert \Psi \Vert \; {\text {with}} \; \sum _k c_k^q < \infty \quad \forall n \in \mathbb {N}\Big \} \;, \end{aligned}$$

where *q* is the Hölder dual of *p*, i.e., $\frac{1}{p} + \frac{1}{q} = 1$. Note that $\mathcal {S}^\otimes _{{\textrm{uni}}} \subset \mathcal {S}^\otimes _q \subset \mathcal {S}^\otimes$.

#### Remark 4.12

It is possible to view the subspace $\prod ^{\otimes C}_{k \in \mathbb {N}} \mathscr {H}_k$ of the equivalence class *C* as the *original Fock space* with respect to the vacuum $\Omega = \prod ^\otimes _{k \in \mathbb {N}} e_{k,0}$: Recall that each $\Psi \in \prod ^{\otimes C}_{k \in \mathbb {N}} \mathscr {H}_k$ can be written in coordinates as (19):

75 $$\begin{aligned} \Psi = \sum _{n(\cdot ) \in F} a(n(\cdot )) \prod ^\otimes _{k \in \mathbb {N}} e_{k,n(k)} \;, \end{aligned}$$

with *F* containing all sequences $(n(k))_{k \in \mathbb {N}}$, such that $n(k)=0$ for almost all *k*. Hence, each $\prod ^\otimes _{k \in \mathbb {N}} e_{k,n(k)}$ is a tensor product state of finitely many particles. Since the Fock norm and the $\widehat{\mathscr {H}}$-norm coincide, the vector $\prod ^\otimes _{k \in \mathbb {N}} e_{k,n(k)}$ can be seen as a Fock space vector normalized to 1. The linear combination (75) with $\sum _{n(\cdot )} |a(n(\cdot ))|^2$ can hence also be interpreted a Fock space vector.

Conversely, each Fock space vector can be written as a countable sequence (75), since the span of the above-mentioned tensor product states is dense in $\mathscr {F}$.

## Implementation: Extended

We proceed with defining implementability of $\mathcal {V}$ by an extended operator $\mathbb {U}_\mathcal {V}$ on $\widehat{\mathscr {H}}$. Lemma 5.3 establishes that $\mathbb {U}_\mathcal {V}$ is well-defined and Lemma 5.4 gives conditions for when $\mathbb {U}_\mathcal {V}$ is an implementer in the extended sense. We then prove our main results by verifying these conditions in Theorem 5.5 for bosons and Theorem 5.6 for fermions.

### Definition of Extended Implementation

The implementer $\mathbb {U}_\mathcal {V}$ is defined on a dense subspace of Fock space $\mathcal {D}_\mathscr {F}\subset \mathscr {F}$, that contains a finite number of particles from the space $\mathcal {D}_{\varvec{f}}$ (defined by (59) with $\varvec{e}= \varvec{f}$):

76 $$\begin{aligned} \mathcal {D}_\mathscr {F}:= {\textrm{span}}\left\{ a^\dagger (\varvec{\phi }_1) \ldots a^\dagger (\varvec{\phi }_N) \Omega , \; N \in \mathbb {N}_0, \; \varvec{\phi }_{\ell } \in \mathcal {D}_{\varvec{f}}\right\} . \end{aligned}$$

The operator $\mathbb {U}_\mathcal {V}$ now maps from $\mathcal {D}_\mathscr {F}$ into an ITP space $\widehat{\mathscr {H}}$. Note that due to Lemma 4.9, the expressions $b^\dagger (\varvec{\phi }):= a^\dagger (u \varvec{\phi }) + a(v \overline{\varvec{\phi }})$ and $b(\varvec{\phi }):= a(u \varvec{\phi }) + a^\dagger (v \overline{\varvec{\phi }})$ define operators $\mathcal {S}^\otimes \rightarrow \mathcal {S}^\otimes$, i.e., in an extended sense.

#### Definition 5.1

We say that a linear operator $\mathbb {U}_\mathcal {V}: \mathcal {D}_\mathscr {F}\rightarrow \widehat{\mathscr {H}}$ **implements** a Bogoliubov transformation $\mathcal {V}$ **in the extended sense**, if for all $\varvec{\phi }\in \mathcal {D}_{\varvec{f}}, \; \Psi \in \mathbb {U}_\mathcal {V}[\mathcal {D}_\mathscr {F}]$, we have that

77 $$\begin{aligned} \mathbb {U}_\mathcal {V}a^\dagger (\varvec{\phi }) \mathbb {U}_\mathcal {V}^{-1} \Psi = b^\dagger (\varvec{\phi }) \Psi \;, \quad \mathbb {U}_\mathcal {V}a(\varvec{\phi }) \mathbb {U}_\mathcal {V}^{-1} \Psi = b(\varvec{\phi }) \Psi \;. \end{aligned}$$

This requires, of course, that $\mathbb {U}_\mathcal {V}^{-1}$ is well-defined. So before establishing (77), we have to show that $\mathbb {U}_\mathcal {V}$ is invertible. This will be one main difficulty within the upcoming proofs.

The implementer $\mathbb {U}_\mathcal {V}$ is constructed as follows: First we define some new vacuum vector $\Omega _\mathcal {V}= \mathbb {U}_\mathcal {V}\Omega \in \mathcal {S}^\otimes$, such that

78 $$\begin{aligned} b(\varvec{\phi }) \Omega _\mathcal {V}= 0. \end{aligned}$$

Then we make $\mathbb {U}_\mathcal {V}$ change $a^\sharp$- into $b^\sharp$-operators:

#### Definition 5.2

Given a Bogoliubov transformed vacuum state $\Omega _\mathcal {V}\in \mathcal {S}^\otimes$, the **Bogoliubov implementer** $\mathbb {U}_\mathcal {V}$ is formally defined on $\mathcal {D}_\mathscr {F}$ by

79 $$\begin{aligned} \mathbb {U}_\mathcal {V}a^\dagger (\varvec{\phi }_1) \ldots a^\dagger (\varvec{\phi }_n) \Omega := b^\dagger (\varvec{\phi }_1) \ldots b^\dagger (\varvec{\phi }_n) \Omega _\mathcal {V}\;, \end{aligned}$$

with $\varvec{\phi }_{\ell } \in \mathcal {D}_{\varvec{f}}$ and $b^\dagger (\varvec{f}_j) = (a^\dagger (u \varvec{f}_j) + a(v \overline{\varvec{f}_j}))$ for all basis vectors $\varvec{f}_j$ in $\varvec{f}$.

#### Lemma 5.3

($\mathbb {U}_\mathcal {V}$ is well-defined) If $\Omega _\mathcal {V}\in \mathcal {S}^\otimes \subseteq \widehat{\mathscr {H}}$ (see (64)), then (79) defines an operator $\mathbb {U}_\mathcal {V}: \mathcal {D}_\mathscr {F}\rightarrow \mathcal {S}^\otimes$.

#### Proof

Both $u \varvec{f}_j$ and $v \overline{\varvec{f}_j}$ are proportional to the same basis vector $\varvec{e}_j$ (bosonic: $\varvec{g}_j$, fermionic: $\varvec{\eta }_j$, see [45]). So the right-hand side of (79) is a finite linear combination of vectors $a^\sharp (\varvec{e}_{j_1}) \ldots a^\sharp (\varvec{e}_{j_n}) \Omega _\mathcal {V}$. Now, $\Omega _\mathcal {V}\in \mathcal {S}^\otimes$ and by Lemma 4.9, each application of $a^\sharp (\varvec{e}_j)$ leaves the vector in $\mathcal {S}^\otimes$. $\square$

#### Lemma 5.4

(Conditions for an implementer $\mathbb {U}_\mathcal {V}$) Suppose that for a Bogoliubov transformation (i.e., $\mathcal {V}$ satisfying (24)) an $\Omega _\mathcal {V}$ satisfying $b(\varvec{\phi }) \Omega _\mathcal {V}= 0$ for all $\varvec{\phi }\in \mathcal {D}_{\varvec{f}} \subseteq \ell ^2$ has been found, such that $\mathbb {U}_\mathcal {V}$ in (79) is well-defined on $\mathcal {D}_\mathscr {F}$ and has an inverse $\mathbb {U}_\mathcal {V}^{-1}$ defined on $\mathbb {U}_\mathcal {V}[\mathcal {D}_\mathscr {F}]$. Then, $\mathbb {U}_\mathcal {V}$ implements $\mathcal {V}$ in the sense of (77) on all $\Psi \in \mathbb {U}_\mathcal {V}[\mathcal {D}_\mathscr {F}]$.

#### Proof

We write $\Psi = \mathbb {U}_\mathcal {V}\Phi$ with $\Phi \in \mathcal {D}_\mathscr {F}$. By linearity, it suffices to prove the statement for $\Phi = a^\dagger (\varvec{\phi }_1) \ldots a^\dagger (\varvec{\phi }_n) \Omega$, which implies by (79) that $\Psi = b^\dagger (\varvec{\phi }_1) \ldots b^\dagger (\varvec{\phi }_n) \Omega _\mathcal {V}$. Checking the first statement of (77), i.e., $\mathbb {U}_\mathcal {V}a^\dagger (\varvec{\phi }) \mathbb {U}_\mathcal {V}^{-1} \Psi = b^\dagger (\varvec{\phi }) \Psi$ is straightforward. For the second statement, we make use of the CAR/CCR of *a*- and *b*-operators, using $\varepsilon = (-1)$ for fermions and $\varepsilon = 1$ for bosons. Here, the CAR/CCR are valid for *a*-operators by definition, and for *b*-operators, since by means of Lemma 4.1, the Bogoliubov relations survive the extension.

80 $$\begin{aligned} \begin{aligned} \mathbb {U}_\mathcal {V}a(\varvec{\phi }) \mathbb {U}_\mathcal {V}^{-1} \Psi&= \sum _{\ell = 1}^n \mathbb {U}_\mathcal {V}a^\dagger (\varvec{\phi }_1) \ldots a^\dagger (\varvec{\phi }_{\ell -1}) \varepsilon ^{\ell +1} \langle \varvec{\phi }, \varvec{\phi }_\ell \rangle a^\dagger (\varvec{\phi }_{\ell +1}) \ldots a^\dagger (\varvec{\phi }_n) \Omega \\&= b(\varvec{\phi }) b^\dagger (\varvec{\phi }_1) \ldots b^\dagger (\varvec{\phi }_n) \Omega _\mathcal {V}- \varepsilon ^{n+1} b^\dagger (\varvec{\phi }_1) \ldots b^\dagger (\varvec{\phi }_n) b(\varvec{\phi }) \Omega _\mathcal {V}\\&\overset{\text {(78)}}{=} b(\varvec{\phi }) b^\dagger (\varvec{\phi }_1) \ldots b^\dagger (\varvec{\phi }_n) \Omega _\mathcal {V}= b(\varvec{\phi }) \Psi \;,\\ \end{aligned} \end{aligned}$$

where we used the convention that the above sums are set to zero for $N = 0$. $\square$

### Bosonic Case

We now show that for a suitable choice of $\Omega _\mathcal {V}$, the operator $\mathbb {U}_\mathcal {V}$ defined in (79) indeed implements the Bogoliubov transformation $\mathcal {V}$.

#### Theorem 5.5

(Implementation via ITP works, bosonic) Consider a bosonic Bogoliubov transformation $$ \mathcal {V}= \left( {\begin{smallmatrix} u &  v \\ \overline{v} &  \overline{u} \end{smallmatrix}} \right) $$ with $v^*v$ having countable spectrum. Let $\widehat{\mathscr {H}} = \prod _{k \in \mathbb {N}}^\otimes \mathscr {H}_k$ be the ITP space (Definition 4.5) with respect to the basis $(\varvec{g}_k)_{k \in \mathbb {N}} \subset \ell ^2$. Define the new vacuum vector

81 $$\begin{aligned} \Omega _\mathcal {V}= \prod _{k \in \mathbb {N}}^\otimes \Omega _{k,\mathcal {V}} := \prod _{k \in \mathbb {N}}^\otimes \left( \left( \left( 1 - \tfrac{\nu _k^2}{\mu _k^2} \right) ^{1/4} \right) \exp {\left( -\tfrac{\nu _k}{2 \mu _k} (a^\dagger (\varvec{g}_k))^2 \right) } \Omega _k \right) \;, \end{aligned}$$

where $\mu _k, \nu _k$ are the singular values of *u*, *v* as in Sect. 3.2. Then, $\mathcal {V}$ is implemented in the sense of (77) by $\mathbb {U}_\mathcal {V}: \mathcal {D}_\mathscr {F}\rightarrow \widehat{\mathscr {H}}$ (79).

#### Proof

By Lemma 5.4, we need to establish the following four points:

1. The new vacuum $\Omega _\mathcal {V}$ is well-defined
2. $\mathbb {U}_\mathcal {V}$ is well-defined on $\mathcal {D}_\mathscr {F}$ (Lemma 5.3 will be used, here)
3. $$ b(\varvec{\phi }) \Omega _\mathcal {V}= 0 $$
4. $$ \mathbb {U}_\mathcal {V}^{-1} $$ exists on $$ \mathbb {U}_\mathcal {V}[\mathcal {D}_\mathscr {F}] $$

$\underline{(1)\,{\mathrm{Well-definedness\,of}}\,\Omega _\mathcal {V}}$: Expression (81) is an ITP of one normalized factor per space $\mathscr {H}_k$. Hence, it is a *C*-sequence, which can be identified with $\Omega _\mathcal {V}\in \widehat{\mathscr {H}}$.

$\underline{(2)\,{\mathrm{Well-definedness\,of}}\,\mathbb {U}_\mathcal {V}}$: follows from Lemma 5.3, if we can establish $\Omega _\mathcal {V}\in \mathcal {S}^\otimes$. By definition of $\mathcal {S}^\otimes$, we need to verify the rapid decay condition $\Vert N_k^n \Omega _\mathcal {V}\Vert \leqslant c_{k,n} \Vert \Omega _\mathcal {V}\Vert$. Since all $\Omega _{k,\mathcal {V}}$ are normalized, this boils down to proving $\Vert N_k^n \Omega _{k,\mathcal {V}} \Vert _k^2 \leqslant c_{k,n}^2$. We explicitly compute

82 $$\begin{aligned} \Vert N_k^n \Omega _{k,\mathcal {V}} \Vert _k^2 = (1-4t^2)^{1/2} \sum _{N = 0}^\infty \frac{t^{2N} (2N)!}{(N!)^2} (2N)^{2n} \;, \end{aligned}$$

with $t = \left| \tfrac{\nu _k}{2 \mu _k} \right| \in [0,1/2)$. Now, the function

83 $$\begin{aligned} N \mapsto \frac{t^{2N} (2N)!}{(N!)^2} (2N)^{2n} \leqslant (2t)^{2N} (2N)^{2n} \;, \end{aligned}$$

is positive, bounded and decays exponentially at $N \rightarrow \infty$ since $0 \leqslant 2t < 1$. So,

84 $$\begin{aligned} \sum _{N = 0}^\infty \frac{t^{2N} (2N)!}{(N!)^2} (2N)^{2n} \leqslant {\text {cons.}} + \sum _{N = 0}^\infty (2t)^{2N} (2N)^{2n} =: c_{k,n}^2 < \infty \;, \end{aligned}$$

which establishes $\Omega _\mathcal {V}\in \mathcal {S}^\otimes$ and hence the claim.

$\underline{(3)\,b(\varvec{\phi })\,{\textrm{annihilates}}\,\Omega _\mathcal {V}}$: This is straightforward to check: Since $\varvec{\phi }\in \mathcal {D}_{\varvec{f}}$, the following sum over *k* is finite:

85 $$\begin{aligned} b(\varvec{\phi }) \Omega _\mathcal {V}=\sum _k \overline{\phi _k} b(\varvec{f}_k) \Omega _\mathcal {V}. \end{aligned}$$

As in the case, where the Shale condition holds, each $b(\varvec{f}_k)$ annihilates the corresponding vacuum vector $\Omega _{k,\mathcal {V}}$, so the finite sum above is 0.

$\underline{(4)\,{\mathrm{Well-definedness\,of}}\,\mathbb {U}_\mathcal {V}^{-1}}$: Consider the basis $\{a^\dagger (\varvec{f}_{k_1}) \ldots a^\dagger (\varvec{f}_{k_N}) \Omega \; \mid \; N \in \mathbb {N}_0, \; k_{\ell } \in \mathbb {N}\}$ of $\mathcal {D}_\mathscr {F}$, where $\varvec{f}_{k_{\ell }}$ are chosen out of the basis $(\varvec{f}_j)_{j \in \mathbb {N}}$ (with $j = k$). If we can show that the set

86 $$\begin{aligned} \{b^\dagger (\varvec{f}_{k_1}) \ldots b^\dagger (\varvec{f}_{k_N}) \Omega _\mathcal {V}\; \mid \; N \in \mathbb {N}_0, \; k_{\ell } \in \mathbb {N}\} \subset \widehat{\mathscr {H}} \;, \end{aligned}$$

with $b^\dagger (\varvec{f}_k) = \mu _k a^\dagger (\varvec{g}_k) + \nu _k a(\varvec{g}_k)$, is linearly independent, we are done, since then ${\textrm{ker}}(\mathbb {U}_\mathcal {V}) = \{0\}$, so $\mathbb {U}_\mathcal {V}$ is injective and hence invertible on its image.

Now, as applications of $b^\dagger _k:= b^\dagger (\varvec{f}_k)$ and $\mathbb {U}_\mathcal {V}$ preserve the ITP structure, it suffices to show that on each mode *k*, the set

87 $$\begin{aligned} \{(b^\dagger _k)^N \Omega _{k,\mathcal {V}} \; \mid \; N \in \mathbb {N}_0 \} \subset \mathscr {H}_k \end{aligned}$$

is linearly independent. But (87) is just the image of the set

88 $$\begin{aligned} \{(a^\dagger (\varvec{f}_k))^N \Omega _k \; \mid \; N \in \mathbb {N}_0 \} \subset \mathscr {F}(\{\varvec{f}_k\}) \end{aligned}$$

under a one-mode Bogoliubov transformation $\mathbb {U}_{k,\mathcal {V}}: \mathscr {F}(\{\varvec{f}_k\}) \rightarrow \mathscr {H}_k$ (defined as $\mathbb {U}_{j,\mathcal {V}}$ in (33)). For a finite number *m* of modes, Bogoliubov transformations can always be implemented by unitary operators, as then the operator $v: \mathbb {C}^m \rightarrow \mathbb {C}^m$ is always Hilbert–Schmidt. Now, (88) is an orthogonal set with no vector being 0, so its image (87) under $\mathbb {U}_{k,\mathcal {V}}$ is also orthogonal with no vector being 0, and hence it is linearly independent. $\square$

### Fermionic Case

#### Theorem 5.6

(Implementation via ITP works, fermionic) Consider a fermionic Bogoliubov transformation $$ \mathcal {V}= \left( {\begin{smallmatrix} u &  v \\ \overline{v} &  \overline{u} \end{smallmatrix}} \right) $$ with $v^*v$ having countable spectrum. Let $\widehat{\mathscr {H}} = \prod _{k \in \mathbb {N}}^\otimes \mathscr {H}_k$ be the ITP space (Definition 4.6). Define the new vacuum vector

89 $$\begin{aligned} \begin{aligned} \Omega _\mathcal {V}=&\prod _{j \in J''}^\otimes \Omega _{j,\mathcal {V}} \otimes \prod _{i \in I'}^\otimes \Omega _{2i,2i-1,\mathcal {V}} \\ :=&\left( \prod _{j \in J''_1}^\otimes a^\dagger (\varvec{\eta }_j) \Omega _j \right) \otimes \left( \prod _{j \in J''_0}^\otimes \Omega _j \right) \otimes \left( \prod _{i \in I'}^\otimes (\alpha _i - \beta _i a^\dagger (\varvec{\eta }_{2i}) a^\dagger (\varvec{\eta }_{2i-1})) \Omega _{2i,2i-1} \right) \;, \end{aligned} \end{aligned}$$

with $\alpha _i, \beta _i$ being the singular values of *u*, *v* as in (40), and with $\Omega _{2i,2i-1}, \Omega _{2i,2i-1,\mathcal {V}} \in \mathscr {H}_{k(i)}$. Then, $\mathcal {V}$ is implemented in the sense of (77) by $\mathbb {U}_\mathcal {V}: \mathcal {D}_\mathscr {F}\rightarrow \widehat{\mathscr {H}}$ (79).

#### Proof

Again, by Lemma 5.4, it suffices to establish the four points in the proof of the bosonic case (Theorem 5.5). Points 1.) and 3.) are analogous, while 2.) follows from Lemma 5.3 as $\mathcal {S}^\otimes = \widehat{\mathscr {H}}$.

$\underline{4.)\,{\mathrm{Well-definedness of}}\,\mathbb {U}_\mathcal {V}^{-1}}$: We proceed as in proof step 4.) in Theorem 5.5. So we are done if we can prove that the set

90 $$\begin{aligned} \{b^\dagger (\varvec{f}_{j_1}) \ldots b^\dagger (\varvec{f}_{j_N}) \Omega _\mathcal {V}\; \mid \; N \in \mathbb {N}_0, \; j_{\ell } \in J \} \subset \widehat{\mathscr {H}} \end{aligned}$$

is linearly independent. This again boils down to proving a linear independence statement on each $\mathscr {H}_k$. The crucial difference now is, that each tensor product factor $\mathscr {H}_k$ may be a Fock space over either one or two modes. We abbreviate $b^\sharp _j:= b^\sharp (\varvec{f}_j)$ and $a^\sharp _j:= a^\sharp (\varvec{\eta }_j)$. For two-mode factors indexed by $i \in I'$, we need to prove linear independence of the set

91 $$\begin{aligned} \{(b^\dagger _{2i})^{N_1} (b^\dagger _{2i-1})^{N_2} \Omega _{k(i),\mathcal {V}} \; \mid \; N_1,N_2 \in \{0, 1\} \} \subset \mathscr {H}_{k(i)} \;. \end{aligned}$$

This follows from $\mathbb {U}_{2i,2i-1,\mathcal {V}}$ (see (42)) being unitary and mapping the set

92 $$\begin{aligned} \{(a^\dagger _{2i})^{N_1} (a^\dagger _{2i-1})^{N_2} \Omega _{k(i)} \; \mid \; N_1,N_2 \in \{0, 1\} \} \subset \mathscr {F}(\{\varvec{f}_{2i}\}) \otimes \mathscr {F}(\{\varvec{f}_{2i-1}\}) \end{aligned}$$

onto (91). For one-mode factors indexed by $j \in J''$ we need linear independence of

93 $$\begin{aligned} \{(b^\dagger _j)^N \Omega _{k(j),\mathcal {V}} \; \mid \; N \in \{0, 1\} \} \subset \mathscr {H}_{k(j)} \;. \end{aligned}$$

This follows again by unitarity of $\mathbb {U}_{j,\mathcal {V}}$, as well as orthogonality and zero-freeness of the set $\{(a^\dagger _j)^N \Omega _{k(j)} \; \mid \; N \in \{0, 1\} \} \subset \mathscr {F}(\{\varvec{f}_j\})$, which is mapped to (93). By linear independence of (91) and (93), we obtain linear independence of (90), which implies injectivity of $\mathbb {U}_\mathcal {V}$ and finishes the proof. $\square$

#### Remark 5.7

We may extend $\mathbb {U}_\mathcal {V}$ to a unitary operator on $\widehat{\mathscr {H}}$: Both in the bosonic and the fermionic case, we have $\widehat{\mathscr {H}} = \prod _{k \in \mathbb {N}}^\otimes \mathscr {H}_k$, compare (60) and (61), where a unitary $\mathbb {U}_{k, \mathcal {V}}: \mathscr {H}_k \rightarrow \mathscr {H}_k$ is defined in (33) (bosonic) and (42) (fermionic). On tensor product states $\Psi ^{(m)}:= \prod _k^\otimes \Psi _k^{(m)}$, we can thus immediately define $\mathbb {U}_\mathcal {V}$ as

94 $$\begin{aligned} \mathbb {U}_\mathcal {V}\Psi ^{(m)} := \prod _k^\otimes (\mathbb {U}_{k, \mathcal {V}} \Psi _k^{(m)}) \;, \qquad \Vert \mathbb {U}_\mathcal {V}\Psi ^{(m)} \Vert = \Vert \Psi ^{(m)} \Vert \;. \end{aligned}$$

By Lemma A.2, there exists an orthonormal set $\{\Psi ^{(m)}\}_{m \in \mathcal {M}}$ such that any $\Psi \in \widehat{\mathscr {H}}$ can be written as a convergent series $\Psi = \sum _{m \in \mathcal {M}} d_m \Psi ^{(m)}$, $d_m \in \mathbb {C}$. One easily checks that $\mathbb {U}_\mathcal {V}$ preserves orthonormality of $\Psi ^{(m)}$. Thus, $\mathbb {U}_\mathcal {V}$ first extends to a bounded operator on all $\Psi$ that are finite linear combinations of $\Psi ^{(m)}$, and then to all $\Psi \in \widehat{\mathscr {H}}$ by continuity. Unitarity of $\mathbb {U}_\mathcal {V}: \widehat{\mathscr {H}} \rightarrow \widehat{\mathscr {H}}$ is then straightforward to check.

#### Remark 5.8

It is crucial that the fermionic ITP space has been chosen as $\widehat{\mathscr {H}} = \prod _k^\otimes \mathscr {H}_k$, with two-mode spaces $\mathscr {H}_k = \mathscr {F}(\{\varvec{\eta }_{2i}\}) \otimes \mathscr {F}(\{\varvec{\eta }_{2i-1}\})$ for Cooper pairs $i \in I'$. If we had just chosen a product of one-mode spaces $\prod _{j \in J}^\otimes \mathscr {F}(\{\varvec{\eta }_j\})$, then invertibility of $\mathbb {U}_\mathcal {V}$ may fail.

As an example, consider a $\mathcal {V}$ with countably infinitely many Cooper pairs $i \in I'$, such that $\alpha _i = \beta _i = \frac{1}{\sqrt{2}}$. Then, each Cooper pair is in the state

95 $$\begin{aligned} \Psi _i := \frac{1}{\sqrt{2}} (| 0 \rangle \otimes | 0 \rangle + | 1 \rangle \otimes | 1 \rangle ) \in \mathbb {C}^4 \;, \end{aligned}$$

i.e., we have a “half particle–hole transformation”. When evaluating the formal ITP $\Omega _\mathcal {V}= \prod _{i \in I'}^\otimes \Psi _i$, we obtain a sum of *C*-sequences: For each pair *i*, one has to choose either $| 0 \rangle \otimes | 0 \rangle$ or $| 1 \rangle \otimes | 1 \rangle$ as a contribution to $\Omega _\mathcal {V}$ and sum over all choices. But now, there are uncountably many such choices, as each corresponds to a binary number of infinitely many digits. And each one gives a contribution of norm $\prod _{i \in I'} \frac{1}{\sqrt{2}} = 0$. So $\Omega _\mathcal {V}= 0$, making $\mathbb {U}_\mathcal {V}$ non-invertible.

## Diagonalization: Extended

As we now have conditions for $\mathcal {V}$ being implementable by $\mathbb {U}_\mathcal {V}$ in the extended sense, it would be interesting to diagonalize quadratic Hamiltonians *H* via $\mathbb {U}_\mathcal {V}$. We now give precise definitions of “quadratic Hamiltonian” and “diagonalized” in the extended sense and provide diagonalizability criteria in Propositions 6.3 and 6.4.

### Definition of Extended Diagonalization

Recall that the extended operator algebra $\overline{\mathcal {A}}_{\varvec{e}}$, defined in (56), consists of all maps *H* that assign to each finite operator product $a^\sharp _{j_1} \ldots a^\sharp _{j_m}$ a complex coefficient $H_{j_1,\ldots , j_m} \in \mathbb {C}$. Each map *H* can be interpreted as a (possibly infinite) sum

96 $$\begin{aligned} H = \sum _m \sum _{j_1,\ldots , j_m} H_{j_1,\ldots , j_m} a^\sharp _{j_1} \ldots a^\sharp _{j_m} \;. \end{aligned}$$

A **formal quadratic Hamiltonian** is an element $H \in \overline{\mathcal {A}}_{\varvec{e}}$, where $H_{j_1,\ldots , j_m} \ne 0$ only appears for $m = 2$ and $H^* = H$. We impose normal ordering on quadratic Hamiltonians (see Remark 6.2), so they read

97 $$\begin{aligned} H = \frac{1}{2} \sum _{j,k \in \mathbb {N}} (2 h_{jk} a^\dagger _j a_k \pm k_{jk} a^\dagger _j a^\dagger _k + \overline{k_{jk}} a_j a_k) \;, \end{aligned}$$

where $\pm$ means $+$ in the bosonic and $-$ in the fermionic case. The term “formal” stresses that *H* is not necessarily an operator on Fock space. To *H* we associate a block matrix

98 $$\begin{aligned} A_H = \begin{pmatrix} h &  \pm k \\ \overline{k} &  \pm \overline{h} \end{pmatrix} \;, \end{aligned}$$

with $h = (h_{jk})_{j,k \in \mathbb {N}}$, $k = (k_{jk})_{j,k \in \mathbb {N}}$ being matrices of infinite size.

Consider a Bogoliubov transformation $$ \mathcal {V}= \left( {\begin{smallmatrix} u &  v \\ \overline{v} &  \overline{u} \end{smallmatrix}} \right) $$. Then, $a^\dagger _j \mapsto b^\dagger _j = \sum _k (u_{jk} a^\dagger _k + \overline{v_{jk}} a_k)$, followed by a **normal ordering**, defines a corresponding algebraic Bogoliubov transformation $\mathcal {V}_{\overline{\mathcal {A}}}: \overline{\mathcal {A}}_{\varvec{e}} \supseteq {\textrm{dom}}(\mathcal {V}_{\overline{\mathcal {A}}}) \rightarrow \overline{\mathcal {A}}_{\varvec{e}}$, where ${\textrm{dom}}(\mathcal {V}_{\overline{\mathcal {A}}}) \subseteq \overline{\mathcal {A}}_{\varvec{e}}$ is a suitable subspace that avoids diverging sums over *k*. The transformed operator and its associated block matrices are

99 $$\begin{aligned} \widetilde{H} = \mathcal {V}_{\overline{\mathcal {A}}}(H) \;, \qquad A_{\widetilde{H}} = \mathcal {V}^* A_H \mathcal {V}\;. \end{aligned}$$

#### Definition 6.1

A formal quadratic Hamiltonian $H \in \overline{\mathcal {A}}_{\varvec{e}}$ is called **diagonalizable in the extended sense** if there exists a Bogoliubov transformation $\mathcal {V}$, such that

100 $$\begin{aligned} \mathcal {V}^* A_H \mathcal {V}= \begin{pmatrix} E &  0 \\ 0 &  \pm E \end{pmatrix} \;, \end{aligned}$$

with $E \geqslant 0$ being Hermitian, $\pm$ being $+$ in the bosonic and $-$ in the fermionic case, and where $\mathcal {V}$ is implementable in the extended sense (see Definition 5.1).

The Hamiltonian associated with $A_{\widetilde{H}}$ is then $\widetilde{H} = {\textrm{d}}\Gamma (E)$, where the matrix *E* provides a positive semidefinite quadratic form on $\mathcal {D}_{\varvec{e}}$. So by Friedrichs’ theorem, it has a self-adjoint extension on ${\textrm{dom}}(E)$. Following [48, Sect. VIII.10], ${\textrm{d}}\Gamma (E)$ is then essentially self-adjoint on $\bigoplus _{n = 0}^\infty {\textrm{dom}}(E)^{\otimes n} \subseteq \mathscr {F}$, so $\widetilde{H}$ defines quantum dynamics on $\mathscr {F}$.

#### Remark 6.2

(Normal ordering constant) Our process of “diagonalizing” a Hamiltonian *H* actually consists of conjugating it with $\mathbb {U}_\mathcal {V}$, so $a^\sharp$ is replaced by $b^\sharp$, *plus a subsequent normal ordering process*. This process is equivalent to adding a constant to the Hamiltonian, namely

101 $$\begin{aligned} c = \frac{1}{2} \left( {\textrm{tr}}(E) - {\textrm{tr}}(h) \right) = \frac{1}{2} \sum _j (E_{jj} - h_{jj}) \;. \end{aligned}$$

The sum might be divergent and hence not a complex number. Nevertheless, using ESS (see Appendix B), we may interpret it as an infinite renormalization constant $c \in {\textrm{Ren}}_1(\mathbb {N})$, namely the one associated with the sequence $c_j = \frac{1}{2} (E_{jj} - h_{jj})$. If *E* now maps $\mathcal {D}_{\varvec{e}}$ into itself (so each column has finitely many non-zero entries), then

$$\begin{aligned} \widetilde{H} = \mathbb {U}_\mathcal {V}^{-1} (H + c) \mathbb {U}_\mathcal {V}\;, \end{aligned}$$

which is in accordance with (2). Otherwise, we may decompose $H = \sum _{n \in \mathbb {N}} H^{(n)}$ and $c = \sum _{n \in \mathbb {N}} c^{(n)}$, such that in $$ \mathcal {V}^* A_{H^{(n)}} \mathcal {V}= \left( {\begin{smallmatrix} E^{(n)} &  0 \\ 0 &  \pm E^{(n)} \end{smallmatrix}} \right) $$, each $E^{(n)}$ maps $\mathcal {D}_{\varvec{e}}$ into itself. So

$$\begin{aligned} \widetilde{H} = \sum _{n \in \mathbb {N}} \mathbb {U}_\mathcal {V}^{-1} (H^{(n)} + c^{(n)}) \mathbb {U}_\mathcal {V}\;, \end{aligned}$$

which is in accordance with (3).

### Bosonic Case

Conditions for the existence of a $\mathcal {V}$, such that $\mathcal {V}^* A_H \mathcal {V}$ is block-diagonal, can be found in [13, Thms. 1 and 4]. We can use them to readily derive conditions for when a formal quadratic Hamiltonian *H* is diagonalizable in the extended sense:

#### Proposition 6.3

(Extended diagonalizability, bosonic case) Let a formal quadratic bosonic Hamiltonian *H* (97) be given such that for the associated block matrix $A_H$ (98) we have $h > 0$, and that $G = h^{-1/2} k h^{-1/2}$ is a bounded operator with $\Vert G \Vert < 1$. Following [13, Theorem 1], there exists a bosonic Bogoliubov transformation $$ \mathcal {V}= \left( {\begin{smallmatrix} u & \quad v \\ \overline{v} & \quad \overline{u} \end{smallmatrix}} \right) $$ such that

102 $$\begin{aligned} \mathcal {V}^* A_H \mathcal {V}= \begin{pmatrix} E & \quad 0 \\ 0 & \quad E \end{pmatrix} \;. \end{aligned}$$

Suppose further that $v^*v$ has countable spectrum.

Then, *H* is diagonalizable in the extended sense.

#### Proof

Consider Definition 6.1 for diagonalizability. The existence of $\mathcal {V}$ as a block matrix associated with a bounded operator on $\ell ^2$ is a direct consequence of [13, Theorem 1].

If the spectrum of $v^*v$ is countable, then implementability of $\mathcal {V}$ in the extended sense follows from Theorem 5.5. $\square$

### Fermionic Case

#### Proposition 6.4

(Extended diagonalizability, fermionic case) Let a formal quadratic fermionic Hamiltonian *H* (97) be given such that for the associated block matrix $A_H$ (98), ${\textrm{dimKer}}(A_H)$ is even or $\infty$. Following [13, Theorem 4], there exists some fermionic Bogoliubov transformation $$ \mathcal {V}= \left( {\begin{smallmatrix} u &  v \\ \overline{v} &  \overline{u} \end{smallmatrix}} \right) $$ such that

103 $$\begin{aligned} \mathcal {V}^* A_H \mathcal {V}= \begin{pmatrix} E & \quad 0 \\ 0 & \quad - E \end{pmatrix} \;. \end{aligned}$$

Suppose further that $v^*v$ has countable spectrum.

Then, *H* is diagonalizable in the extended sense on the ITP space $\widehat{\mathscr {H}}$.

#### Proof

Existence of a unitary $\mathcal {V}$ and of $E \geqslant 0$ follows from [13, Theorem 4]. By unitarity, $\mathcal {V}^* \mathcal {V}= 1 = \mathcal {V}\mathcal {V}^*$, so $\mathcal {V}$ is a fermionic Bogoliubov transformation. If $\sigma (v^*v)$ is countable, then implementability follows from Theorem 5.6. $\square$

## Applications

### Quadratic Bosonic Interaction

Our first example for a quadratic Hamiltonian whose diagonalization requires Bogoliubov transformations “beyond the Shale/Shale–Stinespring condition” is inspired by [25]. We consider a free massive bosonic scalar field, which is interacting by a Wick square $:\phi (\varvec{x})^2:$, with $\phi (\varvec{x}) = a^\dagger (\varvec{x}) + a(\varvec{x})$. We discretize the momentum by putting the system in a box $\varvec{x}\in [-\pi , \pi ]^3$ with periodic boundary conditions. Further, the Wick square is weighted by a real-valued external field $\kappa \in C_c^\infty ([-\pi , \pi ]^3), \kappa (\varvec{x}) \in \mathbb {C}$. The Hamiltonian then reads

104 $$\begin{aligned} \begin{aligned} H&= {\textrm{d}}\Gamma (\varepsilon _{\varvec{p}}) + \frac{1}{2}\int \kappa (\varvec{x}) :\phi (\varvec{x})^2: \; {\textrm{d}}\varvec{x}\\&= \frac{1}{2}\sum _{\varvec{p}_1, \varvec{p}_2 \in \mathbb {Z}^3} (2 \varepsilon _{\varvec{p}_1} \delta (\varvec{p}_1 - \varvec{p}_2) a^\dagger _{\varvec{p}_1} a_{\varvec{p}_2} + 2 \hat{\kappa }(- \varvec{p}_1 + \varvec{p}_2) a^\dagger _{\varvec{p}_1} a_{\varvec{p}_2} + \\&\quad + \hat{\kappa }(- \varvec{p}_1 - \varvec{p}_2) a^\dagger _{\varvec{p}_1} a^\dagger _{\varvec{p}_2} + \hat{\kappa }(\varvec{p}_1 + \varvec{p}_2) a_{\varvec{p}_1} a_{\varvec{p}_2} ) \;, \end{aligned} \end{aligned}$$

with $\hat{\kappa }(\varvec{p}) = \overline{\hat{\kappa }(- \varvec{p})}$ denoting the Fourier transform of $\kappa (\varvec{x})$. For simplicity, we assume that $\kappa (\varvec{x}) = {\mathrm {const.}}$, so we can write $\hat{\kappa }(\varvec{p}) = \kappa \delta (\varvec{p})$, $\kappa \in \mathbb {R}$.

#### Proposition 7.1

For interactions $\kappa > - \frac{m}{2}$ but $\kappa \ne 0$, the Hamiltonian *H* is diagonalizable in the extended sense on $\widehat{\mathscr {H}}$. However, the transformation $\mathcal {V}$ violates the Shale condition, so *H* is not diagonalizable on $\mathscr {F}$.

#### Proof

We directly compute $\mathcal {V}$ and then apply Proposition 6.3. The matrix $A_H$ of *A* is

105 $$\begin{aligned} A_H = \bigoplus _{\varvec{p}\in \mathbb {Z}^3} A_{H,\varvec{p}} \;, \quad A_{H,\varvec{p}} = \begin{pmatrix} h_{\varvec{p}} &  k_{\varvec{p}} \\ k_{\varvec{p}} &  h_{\varvec{p}} \end{pmatrix} \;, \quad h_{\varvec{p}} = (\varepsilon _{\varvec{p}} + \kappa ) \;, \quad k_{\varvec{p}} = \kappa . \end{aligned}$$

We diagonalize all $A_{H,\varvec{p}} \in \mathbb {C}^{2 \times 2}$ separately via

106 $$\begin{aligned} \mathcal {V}= \bigoplus _{\varvec{p}\in \mathbb {Z}^3} \mathcal {V}_{\varvec{p}} \;, \quad \mathcal {V}_{\varvec{p}}^* A_{H,\varvec{p}} \mathcal {V}_{\varvec{p}} = \begin{pmatrix} E_{\varvec{p}} & \quad 0 \\ 0 & \quad E_{\varvec{p}} \end{pmatrix}, \end{aligned}$$

with $E_{\varvec{p}} = \sqrt{h^2 - k^2}$. Following [13, Sect. 1.3], this is done for $|k_{\varvec{p}}| < h_{\varvec{p}}$ by

107 $$\begin{aligned} \mathcal {V}_{\varvec{p}} = \begin{pmatrix} u_{\varvec{p}} & \quad v_{\varvec{p}} \\ \overline{v_{\varvec{p}}} & \quad \overline{u_{\varvec{p}}} \end{pmatrix} \;, \quad u_{\varvec{p}} = c_{\varvec{p}} \;, \quad v_{\varvec{p}} = c_{\varvec{p}} \frac{-G_{\varvec{p}}}{1 + \sqrt{1 - G_{\varvec{p}}^2}} \;, \end{aligned}$$

108 $$\begin{aligned} G_{\varvec{p}} = k_{\varvec{p}} h_{\varvec{p}}^{-1} \;, \quad c_{\varvec{p}} = \sqrt{\frac{1}{2} + \frac{1}{2 \sqrt{1 - G_{\varvec{p}}^2}}}. \end{aligned}$$

Now, $|k_{\varvec{p}}|< h_{\varvec{p}} \quad \Leftrightarrow \quad |\kappa | < \sqrt{|\varvec{p}|^2 + m^2} + \kappa$, which is satisfied for all $\varvec{p}\in \mathbb {Z}^3$, if and only if $\kappa > - \frac{m}{2}$. $h_{\varvec{p}} > 0$ also holds in that case and (106) defines a Bogoliubov transformation $\mathcal {V}$ diagonalizing $A_H$. Regarding Proposition 6.3, $h > 0$ and $\Vert h^{-1/2} k h^{-1/2} \Vert < 1$ follow from $h_{\varvec{p}}$ and $|k_{\varvec{p}}| < h_{\varvec{p}}$ after taking a direct sum. Since $v = \bigoplus _{\varvec{p}\in \mathbb {Z}^3} v_{\varvec{p}}$ can be decomposed into modes, the same holds for $v^*v$, which therefore has countable spectrum. So Proposition 6.3 applies and *H* is diagonalizable in the extended sense on $\widehat{\mathscr {H}}$.

It remains to show that $\mathcal {V}$ violates the Shale condition, i.e., ${\textrm{tr}}(v^*v) = \sum _{\varvec{p}\in \mathbb {Z}^3} |v_{\varvec{p}}|^2 = \infty$. If $|\varvec{p}|$ is large enough (say, $|\varvec{p}|> p_{\max } > 0$), we have $\frac{\kappa d}{|\varvec{p}|} \leqslant G_{\varvec{p}} \leqslant \frac{\kappa }{|\varvec{p}|}$ for any $d < 1$, so

109 $$\begin{aligned} |v_{\varvec{p}}|^2 = \frac{1 + \sqrt{1 - G_{\varvec{p}}^2}}{2 \sqrt{1 - G_{\varvec{p}}^2}} \frac{G_{\varvec{p}}^2}{\left( 1 + \sqrt{1 - G_{\varvec{p}}^2}\right) ^2} \geqslant \frac{\kappa ^2 d^2}{4|\varvec{p}|^2} \;. \end{aligned}$$

We write $\sum _{\varvec{p}} |v_{\varvec{p}}|^2$ as an integral, using indicator functions $\chi _{Q(\varvec{p})}(\cdot )$ of half-open unit cubes $Q(\varvec{p})$ centered at $\varvec{p}= (p_1,p_2,p_3)$:

110 $$\begin{aligned} \sum _{\varvec{p}\in \mathbb {Z}^3} |v_{\varvec{p}}|^2 = \int _{\mathbb {R}^3} f(\varvec{p}') \; {\textrm{d}}\varvec{p}' \;, \quad f(\varvec{p}') = \sum _{\varvec{p}\in \mathbb {Z}^3} |v_{\varvec{p}}|^2 \chi _{Q(\varvec{p})}(\varvec{p}') \;. \end{aligned}$$

Then, for $$ |\varvec{p}'| > p_{\max } $$,

111 $$\begin{aligned} \sum _{\varvec{p}\in \mathbb {Z}^3} |v_{\varvec{p}}|^2 \geqslant \int _{|\varvec{p}| > p_{\max }} f(\varvec{p}') \; {\textrm{d}}\varvec{p}' \geqslant \int _{p_{\max }}^\infty \frac{\kappa ^2 d^2}{4 (|\varvec{p}'| + \frac{\sqrt{3}}{2})^2} 4 \pi |\varvec{p}'|^2 \; {\textrm{d}}|\varvec{p}'| = \infty \;, \end{aligned}$$

where the integral is linearly divergent, which establishes the claim. $\square$

#### Remark 7.2

(Infinite volume case) Here, $\varvec{p}\in \mathbb {R}^3$ and we can construct an analogous $\mathcal {V}$ diagonalizing $A_H$. However, the spectrum of $v^*v$ is then no longer countable.

#### Remark 7.3

(Position-dependent $\kappa (\varvec{x})$) In contrast to [25], we assume a constant interaction strength $\kappa (\varvec{x})$. Physically, it would be desirable to treat any $\kappa \in C_c^\infty$. Then, the decomposition $A_H = \bigoplus _{\varvec{p}\in \mathbb {Z}} A_{H,\varvec{p}}$ fails and it might occur that $v^*v$ has uncountable spectrum.

#### Remark 7.4

(Wick square is not diagonalizable) It may be tempting to set $\varepsilon _{\varvec{p}} = 0$ and to try a diagonalization of only the interaction Hamiltonian $\tfrac{1}{2} \int \kappa (\varvec{x}): \phi (\varvec{x}): \; {\textrm{d}}\varvec{x}$. However, bosonic Wick squares are not diagonalizable by a Bogoliubov transformation, as “the off-diagonal is too large”. For example, on one mode ($\mathfrak {h}\cong \mathbb {C}$), the matrix associated with a Wick square $H_W = 2 a^\dagger a + a^\dagger a^\dagger + a a$ is

112 $$\begin{aligned} A_{H_W} = \begin{pmatrix} h & \quad k \\ \overline{k} & \quad \overline{h} \end{pmatrix} = \begin{pmatrix} 1 & \quad 1 \\ 1 & \quad 1 \end{pmatrix}, \end{aligned}$$

so $\Vert h^{-1/2} k h^{-1/2} \Vert = 1$ and Proposition 6.3 does not apply. However, bosonic Wick products have still been constructed as self-adjoint operators by a suitable GNS construction [49].

### BCS Model

An example with non-implementable fermionic Bogoliubov transformations is the Bardeen–Cooper–Schrieffer (BCS) model for explaining superconductivity [23, 24]. For an overview on recent mathematical advances on BCS theory, we refer the reader to [50, 51] and the references therein. The “Hartree-like approximation” state [23, (2.16)] corresponds to a formal fermionic Bogoliubov vacuum state $\Omega _\mathcal {V}$ as in (89). A mathematical analysis by Haag [24] shows that in the infinite volume limit, the BCS Hamiltonian can indeed be diagonalized by a corresponding Bogoliubov transformation, which is not implementable on Fock space.

We consider a similar model of a fermionic gas inside a box with periodic boundary conditions $\varvec{x}\in [-\pi , \pi ]^3$. Hence, we have discretized momenta $\varvec{p}\in \mathbb {Z}^3$, as well as two spins $s \in \{ \uparrow , \downarrow \}$, leading to a one-particle Hilbert space $\mathfrak {h}= L^2(\mathbb {Z}^3 \times \{ \uparrow , \downarrow \})$. The corresponding Fock space is $\mathscr {F}= \mathscr {F}(\mathbb {Z}^3 \times \{ \uparrow , \downarrow \} )$. We consider the following quadratic Hamiltonian (see [24]), which provides an approximate description for the fermionic gas that becomes exact in the infinite volume limit:

113 $$\begin{aligned} H' = H_0 + H_I' = \sum _{\begin{array}{c} \varvec{p}\in \mathbb {Z}^3 \end{array}} \left( \varepsilon _{\varvec{p}} a_{\varvec{p},\uparrow }^\dagger a_{\varvec{p},\uparrow } + \varepsilon _{\varvec{p}} a_{\varvec{p},\downarrow }^\dagger a_{\varvec{p},\downarrow } - \tilde{\Delta }_{\varvec{p}} a_{\varvec{p},\uparrow }^\dagger a_{\varvec{p},\downarrow }^\dagger + \overline{\tilde{\Delta }_{\varvec{p}}} a_{\varvec{p},\uparrow } a_{\varvec{p},\downarrow } \right) , \end{aligned}$$

with kinetic energy $\varepsilon _{\varvec{p}} = \frac{\varvec{p}^2}{2\,m} - \mu \in \mathbb {R}$ and interaction strength $\tilde{\Delta }_{\varvec{p}} \in \mathbb {C}$, of which we assume $\tilde{\Delta }_{\varvec{p}} \ne 0$. As a basis $(e_j)_{j \in J}$ for identifying $\mathfrak {h}$ with $\ell ^2$, we choose

114 $$\begin{aligned} (e_{\varvec{p},s})_{\begin{array}{c} \varvec{p}\in \mathbb {Z}^3 \\ s \in \{ \uparrow , \downarrow \} \end{array}} \subset L^2(\mathbb {Z}^3 \times \{ \uparrow , \downarrow \}) \;, \quad e_{\varvec{p},s}(\varvec{p}',s') = \delta _{\varvec{p}\varvec{p}'} \delta _{ss'} \;, \end{aligned}$$

with $\delta$ being the Kronecker delta. The corresponding canonical basis of $\ell ^2$ is denoted $(\varvec{e}_{\varvec{p},s})_{\varvec{p}\in \mathbb {Z}^3, s \in \{ \uparrow , \downarrow \} }$. In order to obtain momentum conservation, we have to interpret $a^\dagger _{\varvec{p},\downarrow }, a_{\varvec{p},\downarrow }$ as creating/annihilating a fermion of momentum $- \varvec{p}$. The mode index $\varvec{p}$ is only used for an easier decomposition into modes.

#### Proposition 7.5

The Hamiltonian $H'$ (113) is diagonalizable in the extended sense on $\widehat{\mathscr {H}}$.

#### Proof

We compute $\mathcal {V}$ directly and apply Proposition 6.4. This is done in the block matrix representation $A_{H'} = \bigoplus _{\varvec{p}} A_{H',\varvec{p}}$ and $\mathcal {V}= \bigoplus _{\varvec{p}} \mathcal {V}_{\varvec{p}}$ with

115 $$\begin{aligned} A_{H',\varvec{p}} = \begin{pmatrix} \varepsilon _{\varvec{p}} & \quad 0 & \quad 0 & \quad -\tilde{\Delta }_{\varvec{p}} \\ 0 & \quad \varepsilon _{\varvec{p}} & \quad \tilde{\Delta }_{\varvec{p}} & \quad 0 \\ 0 & \quad \overline{\tilde{\Delta }_{\varvec{p}}} & \quad -\varepsilon _{\varvec{p}} & \quad 0 \\ -\overline{\tilde{\Delta }_{\varvec{p}}} & \quad 0 & \quad 0 & \quad -\varepsilon _{\varvec{p}} \\ \end{pmatrix} \;, \qquad \mathcal {V}_{\varvec{p}} = \begin{pmatrix} u_{\varvec{p}} & \quad 0 & \quad 0 & \quad v_{\varvec{p}} \\ 0 & \quad u_{\varvec{p}} & \quad -v_{\varvec{p}} & \quad 0 \\ 0 & \quad \overline{v}_{\varvec{p}} & \quad \overline{u}_{\varvec{p}} & \quad 0 \\ -\overline{v}_{\varvec{p}} & \quad 0 & \quad 0 & \quad \overline{u}_{\varvec{p}} \\ \end{pmatrix} \;, \end{aligned}$$

where $A_{H',\varvec{p}}, \mathcal {V}_{\varvec{p},s} \in \mathbb {C}^4 \otimes \mathbb {C}^4$. The diagonalized matrix reads

116 $$\begin{aligned} \mathcal {V}_{\varvec{p}}^* A_{H',\varvec{p}} \mathcal {V}_{\varvec{p}} = \begin{pmatrix} E_{\varvec{p}} & \quad 0 & \quad 0 & \quad 0 \\ 0 & \quad E_{\varvec{p}} & \quad 0 & \quad 0 \\ 0 & \quad 0 & \quad -E_{\varvec{p}} & \quad 0 \\ 0 & \quad 0 & \quad 0 & \quad -E_{\varvec{p}} \\ \end{pmatrix} \;, \quad E_{\varvec{p}} = \sqrt{\varepsilon _{\varvec{p}}^2 + |\tilde{\Delta }_{\varvec{p}}|^2} \;, \end{aligned}$$

and the diagonalization is established by

117 $$\begin{aligned} u_{\varvec{p}} = \frac{\tilde{\Delta }_{\varvec{p}}}{\sqrt{(E_{\varvec{p}} - \varepsilon _{\varvec{p}})^2 + |\tilde{\Delta }_{\varvec{p}}|^2 }} \;, \qquad v_{\varvec{p}} = \frac{E_{\varvec{p}} - \varepsilon _{\varvec{p}}}{\sqrt{(E_{\varvec{p}} - \varepsilon _{\varvec{p}})^2 + |\tilde{\Delta }_{\varvec{p}}|^2 }} \;. \end{aligned}$$

From this form, it also follows that ${\textrm{dimKer}}(A_H)$ is either even or $\infty$. So in order to apply Proposition 6.4, we only need to show that the spectrum of $v^*v$ is countable. This is the case, since $v = \bigoplus _{\varvec{p}\in \mathbb {Z}^3} v_{\varvec{p}}$ decays into modes, so also $v^*v = \bigoplus _{\varvec{p}\in \mathbb {Z}^3} (v^*v)_{\varvec{p}}$ decays into modes, where each $(v^*v)_{\varvec{p}}$ is a finite-dimensional matrix with finite spectrum. As the sum over $\varvec{p}$ is countable, also the spectrum of $v^*v$ is countable, and by Proposition 6.4, *H* is diagonalizable on $\widehat{\mathscr {H}}$. $\square$

#### Remark 7.6

(Infinite volume case) The Hamiltonian $H'$ is an approximation to $H = H_0 + H_I$, where $H_I$ is an attractive quartic interaction between fermion pairs. As mentioned above, this approximation is only exact in the infinite volume limit. In that case, $\varvec{p}\in \mathbb {R}^3$ and (115)–(117) still yield a Bogoliubov transformation $\mathcal {V}$ diagonalizing $A_{H'}$. However, $v^*v$ has generally uncountable spectrum, so Theorem 5.6 does not apply.

## Data Availability

No datasets were generated during this work.

## Results on the Infinite Tensor Product Space

Our first proposition characterizes when exactly two *C*-sequences correspond to the same functional $\Psi \in \widehat{\mathscr {H}} = \prod _{k \in I}^\otimes \mathscr {H}_k$. This allows us to define operators on $\widehat{\mathscr {H}}$ in Sect. 4.2. Recall that any *C*-sequence $(\Psi ) = (\Psi _k)_{k \in I}$ gives rise to a unique functional $\Psi \in \widehat{\mathscr {H}}$ by the embedding $\Psi = \iota ((\Psi ))$.

### Proposition A.1

Whenever $(\Psi ), (\Psi ') \in {\textrm{Cseq}}$ represent the same functional $\Psi = \Psi '$, then there exists a family of complex numbers $(c_k)_{k \in I}$ such that

118 $$\begin{aligned} \Psi _k = c_k \Psi _k' \quad \forall k \in I \;, \quad \text {and} \quad \prod _{k \in I} c_k = 1 \;, \end{aligned}$$

using the notion of convergence for an infinite product from Sect. 2.2.

Conversely, if $(\Psi ), (\Psi ') \in {\textrm{Cseq}}$ just differ by a family $(c_k)_{k \in I}$ as in (118), then they represent the same functional $\Psi = \Psi '$.

### Proof

For the first statement, we must prove that $\Psi _k$ and $\Psi _k'$ are parallel for any $k \in I$. So let us fix a *k* and decompose $\Psi _k = \Psi _k^\parallel + \Psi _k^\perp$ with $\Psi _k^\parallel \parallel \Psi _k'$ and $\Psi _k^\perp \perp \Psi _k'$, and suppose that $\Psi _k^\perp \ne 0$. Now, choose some *C*-sequences $(\Phi ), (\Phi ')$, that agree on all $k' \ne k$ and with $\Phi _k \parallel \Psi _k$ and $\Phi '_k \parallel \Psi '_k$, as well as $\Vert \Phi _k \Vert _k = \Vert \Phi '_k \Vert _k = 1$. Then,

119 $$\begin{aligned} \begin{aligned} \langle \Psi , \Phi ' \rangle =&\langle \Psi _k, \Phi _k' \rangle _k \prod _{k \ne k'} \langle \Psi _{k'}, \Phi '_{k'} \rangle _{k'} = \Vert \Psi _k^\parallel \Vert _k \prod _{k \ne k'} \langle \Psi _{k'}, \Phi _{k'} \rangle _{k'}\\ <&\Vert \Psi _k \Vert _k \prod _{k \ne k'} \langle \Psi _{k'}, \Phi _{k'} \rangle _{k'} = \langle \Psi _k, \Phi _k \rangle _k \prod _{k \ne k'} \langle \Psi _{k'}, \Phi _{k'} \rangle _{k'} = \langle \Psi , \Phi \rangle \;. \end{aligned} \end{aligned}$$

By the same arguments, $\langle \Psi ', \Phi \rangle < \langle \Psi ', \Phi ' \rangle$. But since $(\Psi ), (\Psi ')$ correspond to the same functional $\Psi = \Psi '$, we can freely exchange both expressions within the scalar product:

120 $$\begin{aligned} \langle \Psi , \Phi ' \rangle = \langle \Psi ', \Phi ' \rangle > \langle \Psi ', \Phi \rangle = \langle \Psi , \Phi \rangle \;. \end{aligned}$$

This contradicts (119) and thus establishes $\Psi _k = c_k \Psi _k'$. Convergence of $\prod _{k \in I} c_k$ can be seen as follows: We have

121 $$\begin{aligned} \Vert \Psi \Vert ^2 = \langle \Psi ', \Psi \rangle = \prod _{k \in I} \langle \Psi '_k, c_k \Psi '_k \rangle _k = \prod _{k \in I} c_k \Vert \Psi '_k \Vert _k^2 \;. \end{aligned}$$

So if $\prod _{k \in I} c_k$ was not convergent, i.e., $\sum _k |c_k - 1| = \infty$, then for the product on the right-hand side, we would have

122 $$\begin{aligned} \sum _k \left| c_k \Vert \Psi '_k \Vert _k^2 - 1 \right| \geqslant \underbrace{\sum _k \Vert \Psi '_k \Vert _k^2 \left| c_k - 1 \right| }_{(*)} - \underbrace{\sum _k \left| \Vert \Psi '_k \Vert _k^2 - 1 \right| }_{< \infty } \;. \end{aligned}$$

Now, $\Vert \Psi '_k \Vert _k^2 > 1/2$ for all but finitely many *k*, so $(*)$ and thus the first expression in (122) diverges. This is a contradiction to (121) being convergent. So $\prod _{k \in I} c_k$ indeed yields a complex number.

But since $\Vert \Psi \Vert ^2 = \Vert \Psi ' \Vert ^2 = \prod _{k \in I} \Vert \Psi '_k \Vert _k^2$, we immediately obtain $\prod _{k \in I} c_k = 1$ from (121).

The converse statement can readily be seen by computing the action of the functionals $\Psi , \Psi '$ on some $\Phi \in {\textrm{Cseq}}$:

123 $$\begin{aligned} \langle \Psi , \Phi \rangle = \prod _{k \in I} \langle \Psi _k, \Phi _k \rangle _k = \prod _{k \in I} \langle c_k \Psi '_k, \Phi _k \rangle _k = \left( \prod _{k \in I} \overline{c_k} \right) \prod _{k \in I} \langle \Psi '_k, \Phi _k \rangle _k = \langle \Psi ', \Phi \rangle \;. \end{aligned}$$

$$\square $$

By [3, Lemma 4.1.1], all subspaces $\prod ^{\otimes C}_{k \in I} \mathscr {H}_k$ of $\widehat{\mathscr {H}} = \prod ^\otimes _{k \in I} \mathscr {H}_k$ are **mutually orthogonal**. This allows for a particularly simple decomposition.

### Lemma A.2

For any $\Psi \in \widehat{\mathscr {H}}$, we can write

124 $$\begin{aligned} \Psi = \sum _{m \in \mathcal {M}} d_m \Psi ^{(m)} = \sum _{m \in \mathcal {M}} d_m \prod ^\otimes _{k \in I} \Psi ^{(m)}_k \;, \end{aligned}$$

with $\mathcal {M}$ being a subset of $\mathbb {N}$, $\Psi ^{(m)}$ defined by the mutually orthogonal $C_0$-sequences $(\Psi ^{(m)})$ with $\Vert \Psi ^{(m)} \Vert = 1$ and where $\sum _m |d_m|^2 < \infty$ is a complex sequence.

Moreover, one can choose a fixed set $Z = \{\Psi ^{(a)}\}_{a \in A}$ defined by mutually orthogonal, normalized $C_0$-sequences $(\Psi ^{(a)})$, such that for all $\Psi \in \widehat{\mathscr {H}}$, the form (124) can be achieved by taking only $\Psi ^{(m)} \in Z$. The decomposition (124) is then unique up to the choice of the $\Psi ^{(m)}_k$ representing $\Psi ^{(m)}$.

So *Z* is an orthonormal basis of $\widehat{\mathscr {H}}$ that might be uncountable, but the elements $\Psi \in \widehat{\mathscr {H}}$ are all countable linear combinations with coefficient sequences in $\ell ^2$.

### Proof

By definition, any $\Psi \in \widehat{\mathscr {H}}$ can be approximated by a Cauchy sequence $(\Psi ^{(r)})_{r \in \mathbb {N}} \subseteq \widetilde{\prod }^{\otimes }_{k \in I} \mathscr {H}_k, \Psi ^{(r)} \rightarrow \Psi$. So each $\Psi ^{(r)}$ can be written as a finite linear combination of *C*-sequences. All *C*-sequences, that are no $C_0$-sequences, must have norm 0, so we drop them and simply write

125 $$\begin{aligned} \Psi ^{(r)} = \sum _{\ell = 1}^{L_r} \Psi ^{(r)}_{\ell } \;, \end{aligned}$$

with $(\Psi ^{(r)}_{\ell })$ being $C_0$-sequences. Now, the $C_0$-sequences decay into mutually orthogonal equivalence classes *C*, out of which countably many are occupied by any $\Psi ^{(r)}$. So

126 $$\begin{aligned} \Psi ^{(r)} = \sum _C \sum _{\ell :(\Psi ^{(r)}_{\ell }) \in C} \Psi ^{(r)}_{\ell } =: \sum _{C} \Psi ^{(r)}_C \;. \end{aligned}$$

By orthogonality of the subspaces $\Psi \in \prod ^{\otimes C}_{k \in I} \mathscr {H}_k$, we have

127 $$\begin{aligned} \Vert \Psi ^{(r)} - \Psi ^{(s)} \Vert ^2 = \sum _C \Vert \Psi ^{(r)}_C - \Psi ^{(s)}_C \Vert ^2 \;. \end{aligned}$$

$(\Psi ^{(r)})_{r \in \mathbb {N}}$ is a Cauchy sequence, so $(\Psi ^{(r)}_C)_{r \in \mathbb {N}}$ is also a Cauchy sequence for all *C*. That means, the limit $\Psi _C = \lim _{r \rightarrow \infty } \Psi ^{(r)}_C$ exists and by orthogonality of the $\Psi ^{(r)}_C$ for each *r*,

128 $$\begin{aligned} \lim _{r \rightarrow \infty } \sum _C \Psi ^{(r)}_C = \sum _C \lim _{r \rightarrow \infty } \Psi ^{(r)}_C \qquad \Leftrightarrow \qquad \Psi = \sum _C \Psi _C \;. \end{aligned}$$

We may now write $\Psi _C$ in coordinates:

129 $$\begin{aligned} \Psi _C = \lim _{r \rightarrow \infty } \Psi ^{(r)}_C = \lim _{r \rightarrow \infty } \sum _{\ell :(\Psi ^{(r)}_{\ell }) \in C} \Psi ^{(r)}_{\ell } = \sum _{n(\cdot ) \in F} a_C(n(\cdot )) \prod ^\otimes _{k \in I} e_{k,n(k)} \;, \end{aligned}$$

so also $\Psi$ can be written as a countable sum over mutually orthogonal, normalized $C_0$-sequences with coordinates $a_C(n(\cdot ))$. We index the sequences and coordinates by $(\Psi ^{(m)})$ and $d_m$, which yields the desired form (124).

Square summability of the $d_m$ can be seen by

130 $$\begin{aligned} \sum _m |d_m|^2 = \sum _C \sum _{n(\cdot ) \in F} |a_C(n(\cdot ))|^2 \;. \end{aligned}$$

Now, the set $Z = \{ \Psi ^{(a)} \}_{a \in A}$ is exactly the union of all vectors $\prod ^\otimes _{k \in I} e_{k,n(k)}$ over all classes *C*, which is indeed a set of mutually orthogonal, normalized vectors.

Uniqueness of the decomposition follows by orthogonality of the $\Psi ^{(r)}$. $\square$

Further, by [3, Definition 6.1.1], two $C_0$-sequences $(\Psi ), (\Phi )$ are weakly equivalent, if and only if there exists a family $(z_k)_{k \in I} \subseteq \mathbb {C}$ with $(z_k \Psi _k)_{k \in I}$ being (strongly) equivalent to $(\Phi _k)_{k \in I}$. From that, we conclude:

### Lemma A.3

Let $C_w$ be the weak equivalence class of a $C_0$-sequence $(\Phi ) = (\Phi _k)_{k \in I}$, choose an orthonormal basis $(e_{k,n})_{n \in \mathbb {N}_0}$ for each $\mathscr {H}_k$ such that $\Phi _k = c_{k,0}$ and define for any $C_0$-sequence $(\Psi ) = (\Psi _k)_{k \in I}$ the coordinates $c_{k,n}:= \langle e_{k,n}, \Psi _k \rangle _k$.

Then, $\prod ^{\otimes C_w}_{k \in I} \mathscr {H}_k$ is exactly the closure of the span of all normalized $C_0$-sequences, where $|c_{k,0}| = 1$ for all but finitely many $k \in I$.

Note that the last statement means $c_{k,n} = 0$ for $n \geqslant 1$ and for those *k*. In simple words, Lemma A.3 asserts that replacing *C* by $C_w$ in the equivalence class is done via replacing $c_{k,0} = 1$ by $|c_{k,0}| = 1$.

### Proof

First, we prove that any normalized $C_0$-sequence $(\Psi )$ with $|c_{k,0}| = 1$ almost everywhere is weakly equivalent to $(\Phi )$: We can define a family of phase rotations $|z_k| = 1$, such that $z_k c_{k,0} = 1$ for all *k* with $|c_{k,0}| = 1$. So $(z_k \Psi _k)_{k \in I}$ has $z_k c_{k,0} = 1$ almost everywhere and is hence a $C_0$-sequence strongly equivalent to $(\Phi )$. Therefore, $(\Psi )$ is weakly equivalent to $(\Phi )$. So $\Psi \in \prod ^{\otimes C_w}_{k \in I} \mathscr {H}_k$ and the same holds for the span of these $C_0$-sequences and their closure with respect to the Hilbert space topology on $\widehat{\mathscr {H}}$.

Conversely, any $\Psi \in \prod ^{\otimes C_w}_{k \in I} \mathscr {H}_k$ is within the closure of the span of normalized $C_0$-sequences with $|c_{k,0}| = 1$ almost everywhere: By Lemma A.2, we may write

$$\begin{aligned} \Psi = \sum _{m \in \mathcal {M}} d_m \prod ^\otimes _{k \in I} \Psi ^{(m)}_k \;, \end{aligned}$$

where $\sum _m |d_m|^2 < \infty$ and the $(\Psi ^{(m)}) = (\Psi ^{(m)}_k)_{k \in I}$ with $\Vert \Psi ^{(m)} \Vert = 1$ are orthogonal. Further, we may choose $(\Psi ^{(m)}) \sim _w (\Phi )$, since all $(\Psi ^{(m)})$ were constructed to come from a (strong) equivalence class *C* contained within $C_w$. So there exist families $(z^{(m)}_k)_{k \in I}, \; |z^{(m)}_k| = 1$, such that $(z^{(m)}_k \Psi ^{(m)}_k)_{k \in I} \sim (\Phi )$ for all $m \in \mathcal {M}$. By strong equivalence, we may approximate each $(z^{(m)}_k \Psi ^{(m)}_k)$ up to arbitrary precision $\varepsilon > 0$ by a linear combination of (normalized) families $\Psi ^{(m,\varepsilon )}$, such that, when writing these families in coordinates, we have $c^{(m,\varepsilon )}_{k,0} = 1$ almost everywhere in $k \in I$. Hence, the families $((z^{(m)}_k)^{-1} \Psi ^{(m,\varepsilon )}_k)_{k \in I}$ approximate $\Psi ^{(m)}$ up to precision $\varepsilon$. They satisfy $|(z^{(m)}_k)^{-1} c^{(m,\varepsilon )}_{k,0}| = 1$ almost everywhere, so $\Psi ^{(m)}$ can be approximated up to arbitrary precision by a linear combination of $C_0$-sequences with the above-mentioned property. And by (124) and convergence of $|d_m|^2$, also an arbitrary approximation of $\Psi$ is possible by linear combinations of normalized $C_0$-sequences with $|c_{k,0}| = 1$ almost everywhere. $\square$

## Extended State Space

As mentioned in the introduction, it is also possible to achieve results similar to Theorems 5.5 and 5.6 in the “Extended State Space” setting from [26, 27]. In this appendix, we define extensions $\overline{\mathscr {F}}, \overline{\mathscr {F}}_{{\textrm{ex}}}$ adapted to $v^* v$ having discrete spectrum, so the definitions are simpler than in [26, 27].

### ESS Construction

We start by defining

- The space of **generalized one-particle wave functions**
  131 $$\begin{aligned} \mathcal {E}= \mathcal {E}(\mathbb {N}) = \mathbb {C}^\mathbb {N}:= \{ \varvec{\phi }: \mathbb {N}\rightarrow \mathbb {C}\}, \end{aligned}$$
  that is, the space of complex sequences $\varvec{\phi }= (\phi _j)_{j \in \mathbb {N}}$. It extends $\mathcal {D}_{\varvec{g}}$ (bosonic) or $\mathcal {D}_{\varvec{\eta }}$ (fermionic) and replaces the smooth function space $\dot{\mathscr {S}}^\infty _1$ from [26].
- The space of **generalized** *N***-particle wave functions**
  132 $$\begin{aligned} \mathcal {E}^{(N)}(\mathbb {N}) := \{ \Psi : \mathbb {N}^N \rightarrow \mathbb {C}\} \;. \end{aligned}$$
- The space of **generalized Fock space functions** (which replaces $\dot{\mathscr {S}}^\infty _\mathscr {F}$ from [26]):
  133 $$\begin{aligned} \mathcal {E}_\mathscr {F}(\mathbb {N}) := \bigoplus _{N = 0}^\infty \mathcal {E}^{(N)}(\mathbb {N}) = \{ \Psi : \mathcal {Q}(\mathbb {N}) \rightarrow \mathbb {C}\} \;. \end{aligned}$$

$\mathcal {E}_\mathscr {F}$ is *not* yet the final extended state space. Formal state vectors occurring in QFT include products of functions $\Psi _m \in \mathcal {E}_\mathscr {F}$ and exponentials of divergent sums $e^\mathfrak {r}$ with “$\mathfrak {r}= \pm \infty$”, see [26]. Hence, we need to construct structures accommodating such infinite quantities.

First, we introduce a **space of renormalization factors** ${\textrm{Ren}}_1(\mathbb {N})$, whose elements are sequences that represent formal (and possibly divergent) series, thus replacing the formal (and possibly divergent) integrals from ${\textrm{Ren}}_1$ in [26].

- ${\textrm{Ren}}_1(\mathbb {N}):= \mathcal {E}/_{\sim _{{\textrm{Ren}}_1}}$. Here, for $\varvec{r}_1, \varvec{r}_2 \in \mathcal {E}(\mathbb {N})$, we define $\varvec{r}_1 \sim _{{\textrm{Ren}}_1} \varvec{r}_2$ if and only if $(\varvec{r}_1 - \varvec{r}_2) \in \ell ^1 = L^1(\mathbb {N})$ and $\sum _{j \in \mathbb {N}} (r_{1,j} - r_{2,j}) = 0$.

We denote elements of ${\textrm{Ren}}_1$ by $\mathfrak {r}= [\varvec{r}]$ and identify $\mathfrak {r}= \sum _{j \in \mathbb {N}} r_j \in \mathbb {C}$, if $\varvec{r}\in \ell ^1$. All ${\textrm{Ren}}_1$-elements not identifiable with a $\mathbb {C}$-number can be thought of as “controlled infinitely large numbers”.

Multiplication of ${\textrm{Ren}}_1$-elements is allowed by introducing the free vector spaces ${\textrm{Pol}}_P(\mathbb {N})$, $P \in \mathbb {N}$, called space of **renormalization polynomials of degree** *P*, and defined as being spanned by all commutative products $\mathfrak {r}_1 \cdot \ldots \cdot \mathfrak {r}_p, \; p \leqslant P, \; \mathfrak {r}_j \in {\textrm{Ren}}_1(\mathbb {N})$. Again, we identify equivalent terms by modding out an equivalence relation:

- ${\textrm{Ren}}_P(\mathbb {N}):= {\textrm{Pol}}_P(\mathbb {N}) /_{\sim _{{\textrm{Ren}}_P}}$ with $\sim _{{\textrm{Ren}}_P}$ generated by both $\mathfrak {r}_1 \mathfrak {r}_2 \ldots \mathfrak {r}_p \sim _{{\textrm{Ren}}_P} c_1 \mathfrak {r}_2 \ldots \mathfrak {r}_p$ for $\mathfrak {r}_1 = \sum _{j \in \mathbb {N}} r_{1,j} = c_1 \in \mathbb {C}$ and $(c_1 c_2) \mathfrak {r}_1\ldots \mathfrak {r}_p \sim _{{\textrm{Ren}}_P} c_1 (c_2 \mathfrak {r}_1) \ldots \mathfrak {r}_p$.

The space of **renormalization polynomials** is then given by

134 $$\begin{aligned} {\textrm{Ren}}(\mathbb {N}) := \bigcup _{P \in \mathbb {N}} {\textrm{Ren}}_P(\mathbb {N}) \;. \end{aligned}$$

Wave function renormalization factors usually take the form $e^\mathfrak {r}$, where $\mathfrak {r}\in {\textrm{Ren}}_1$ may or may not correspond to a finite number, as well as linear combinations of such terms $e^\mathfrak {r}$. We will interpret them as elements of a suitably defined field ${\textrm{eRen}}$. For this, consider the group algebra $\mathbb {C}[{\textrm{Ren}}_1]$, where the group is $({\textrm{Ren}}_1, +)$ with addition as described above. We identify elements $\mathfrak {r}\in {\textrm{Ren}}_1$ with the symbolic expression $e^{\mathfrak {r}}$, both to distinguish the two additions in $({\textrm{Ren}}_1, +)$ and $\mathbb {C}[{\textrm{Ren}}_1]$, as well as to be congruent with the intuition that $e^{\mathfrak {r}}$ is an exponential of a finite or infinite number. Elements in $\mathbb {C}[{\textrm{Ren}}_1]$ are then denoted as $c_1 e^{\mathfrak {r}_1} + \ldots + c_M e^{\mathfrak {r}_M}, \; c_j \in \mathbb {C}, \; \mathfrak {r}_j \in {\textrm{Ren}}_1$. Some elements of $\mathbb {C}[{\textrm{Ren}}_1]$ are intuitively equal to zero. We set them equivalent to zero by modding out an ideal $\mathcal {I}\subset \mathbb {C}[{\textrm{Ren}}_1]$ generated by all elements $e^c e^\mathfrak {r}- e^{c + \mathfrak {r}}$ with $c \in \mathbb {C}, \; \mathfrak {r}\in {\textrm{Ren}}_1$.

As in [26, Proposition 3.2] one proves that the quotient ring $\mathbb {C}[{\textrm{Ren}}_1]/\mathcal {I}$ has no proper zero divisors. So the following quotient field exists, which is a field extension of $\mathbb {C}$:

- ${\textrm{eRen}}(\mathbb {N}):= \{ \mathfrak {c}= a_1 / a_2 \; \mid \; a_1, a_2 \in \mathbb {C}[{\textrm{Ren}}_1(\mathbb {N})]/\mathcal {I}\}$ is the **field of wave function renormalizations**.

The formal state vectors appearing in QFT are of the form $\Psi = \sum _m \mathfrak {c}_m \Psi _m$ with $\mathfrak {c}_m \in {\textrm{eRen}}$ and $\Psi _m \in \mathcal {E}_\mathscr {F}$. Those can be described as elements of the following space:

- $\overline{\mathscr {F}}(\mathbb {N}):= \overline{\mathscr {F}}_0(\mathbb {N}) /_{\sim _{\textrm{F}}}$ is the **first extended state space**, where $\overline{\mathscr {F}}_0(\mathbb {N})$ is the free ${\textrm{eRen}}$-vector space over $\mathcal {E}_\mathscr {F}$ (all finite ${\textrm{eRen}}$-linear combinations) and $\sim _{\textrm{F}}$ is generated by $(c \mathfrak {c}) \Psi _m \sim _{\textrm{F}} \mathfrak {c}(c \Psi _m)$ for $c \in \mathbb {C}$.

For intermediate calculations, we will need an even larger vector space $\overline{\mathscr {F}}_{{\textrm{ex}}}$, which allows for multiplication of $\Psi$ by elements of ${\textrm{Ren}}$. We define ${\textrm{Ren}}^\mathcal {Q}(\mathbb {N})$ to consist of all functions $\mathcal {Q}(\mathbb {N}) \rightarrow {\textrm{Ren}}$, which replaces the similarly-defined ${\textrm{Ren}}^{\dot{\mathcal {Q}}}$ from [26].

- $\overline{\mathscr {F}}_{{\textrm{ex}}}(\mathbb {N}):= \overline{\mathscr {F}}_{{\textrm{ex}},0}(\mathbb {N}) /_{\sim _{\textrm{Fex}}}$ is the **second extended state space**, where $\overline{\mathscr {F}}_{{\textrm{ex}},0}(\mathbb {N})$ is the set of all countable ${\textrm{eRen}}$-linear combinations $\Psi = \sum _{m \in \mathbb {N}} \mathfrak {c}_m \Psi _m$ with $\mathfrak {c}_m \in {\textrm{eRen}}(\mathbb {N}), \Psi _m \in {\textrm{Ren}}^\mathcal {Q}(\mathbb {N})$ and where $\sim _{\textrm{Fex}}$ is generated by $(c \mathfrak {c}) \Psi _m \sim _{\textrm{Fex}} \mathfrak {c}(c \Psi _m)$ for $c \in \mathbb {C}$.

We may embed the complex numbers into ${\textrm{Ren}}$ by identifying $c \in \mathbb {C}$ with $c e^0 \in {\textrm{eRen}}$. Hence, the space $\overline{\mathscr {F}}$ can be embedded into $\overline{\mathscr {F}}_{{\textrm{ex}}}$.

#### Remark B.1

(Comparison with non-standard analysis) The construction of ${\textrm{Ren}}_1$ might remind about that of the extended real line ^*^ $\mathbb {R}$ in non-standard analysis [52, Sect. 1.1], since both extend $\mathbb {R}$ by modding out an equivalence relation on sequences. One may therefore ask whether ${\textrm{Ren}}_1$ can be naturally embedded into ^*^ $\mathbb {R}$ or vice versa. This is not the case. First, note that ${\textrm{Ren}}_1$ extends $\mathbb {C}$ and not just $\mathbb {R}$, while there are no elements corresponding to complex numbers in ^*^ $\mathbb {R}$. Even when restricting to real sequences, an embedding would require to identify a sequence $\varvec{r}= (r_j)_{j \in \mathbb {N}}$, $[\varvec{r}] \in {\textrm{Ren}}_1$, with its series $\varvec{s}(\varvec{r}):= (s(\varvec{r})_j)_{j \in \mathbb {N}}$, $s(\varvec{r})_j:= \sum _{\ell = 1}^j r_\ell$ with respect to ^*^ $\mathbb {R}$. This is needed to be consistent with the identification $\varvec{r}= c \in \mathbb {R}$ in ${\textrm{Ren}}_1$ whenever $\sum _j r_j = c < \infty$, as well as the identification $\varvec{s}(\varvec{r}) = c \in \mathbb {R}$ in ^*^ $\mathbb {R}$, whenever $s(\varvec{r})_j = c$ for all $j > J$, $J \in \mathbb {N}$.

Now, there always exists an infinite set $A \subset \mathbb {N}$, such that $\varvec{s}(\varvec{r}_1)$ and $\varvec{s}(\varvec{r}_2)$ equivalent with respect to ^*^ $\mathbb {R}$ whenever they agree on $\mathbb {N}\setminus A$. However, the difference between $\varvec{r}_1$ and $\varvec{r}_2$ on *A* may not be $\ell ^1$-summable, rendering the two sequences inequivalent with respect to ${\textrm{Ren}}_1$. So ${\textrm{Ren}}_1$ cannot be naturally embedded into ^*^ $\mathbb {R}$.

Conversely, ^*^ $\mathbb {R}$ contains many inequivalent $\varvec{s}(\varvec{r})$ converging to the same $c \in \mathbb {R}$ (corresponding to infinitesimals), even with $\ell ^1$-summable differences, while the corresponding $\varvec{r}$ are all equivalent with respect to ${\textrm{Ren}}_1$.

### Operator Lift and Implementation

Creation and annihilation operators $a^\dagger (\varvec{\phi }), a(\varvec{\phi })$ are defined on $\Psi _m: \mathcal {Q}(\mathbb {N}) \rightarrow \mathbb {C}$ in similarity to (10). Formally,

135 $$\begin{aligned} (a_\pm ^\dagger (\varvec{\phi }) \Psi _m)(q) = \sum _{k = 1}^N \frac{(\pm 1)^k}{\sqrt{N}} \phi _{j_k} \Psi _m(q \setminus j_k) \;, \qquad (a_\pm (\varvec{\phi }) \Psi _m)(q) = \sqrt{N+1} \sum _j \overline{\phi _j} \Psi _m(q,j) \;. \end{aligned}$$

Using that there is a natural distribution pairing between $\mathcal {D}$ and $\mathcal {E}$, one easily verifies the following well-definedness statement.

#### Lemma B.2

(Products of $a^\dagger , a$ are well-defined on the ESS) Consider the ESS $\overline{\mathscr {F}}$ built over $\mathcal {E}$. Then, (135) uniquely defines operators $a^\dagger (\varvec{\phi }), a(\varvec{\phi })$ as follows:

136 $$\begin{aligned} \begin{aligned}&a^\dagger (\varvec{\phi }): \overline{\mathscr {F}}\rightarrow \overline{\mathscr {F}}\;, \quad  &   a(\varvec{\phi }): \overline{\mathscr {F}}\rightarrow \overline{\mathscr {F}}  &   \quad \forall \varvec{\phi }\in \mathcal {D}\;, \\&a^\dagger (\varvec{\phi }): \overline{\mathscr {F}}\rightarrow \overline{\mathscr {F}}\;, \quad  &   a(\varvec{\phi }): \overline{\mathscr {F}}\rightarrow \overline{\mathscr {F}}_{{\textrm{ex}}}  &   \quad \forall \varvec{\phi }\in \mathcal {E}. \end{aligned} \end{aligned}$$

Note that the CAR/CCR are a direct consequence of (135) and hence still valid for the operator extensions.

The condition for $\mathbb {U}_\mathcal {V}: \mathcal {D}_\mathscr {F}\rightarrow \overline{\mathscr {F}}$ being an “extended implementer” is identical to the ITP case (Definition 5.1). Also Definition 5.2 for $\mathbb {U}_\mathcal {V}$, given a Bogoliubov vacuum $\Omega _\mathcal {V}\in \overline{\mathscr {F}}$, carries over from ITP to ESS. Then, Lemma 5.3 (well-definedness of $\mathbb {U}_\mathcal {V}$) and Lemma 5.4 are established analogously. By checking the four conditions as in the proofs of Theorems 5.5 and 5.6, it is then straightforward to verify the following two results.

#### Proposition B.3

(Implementation via ESS works, bosonic) Consider a bosonic Bogoliubov transformation $$ \mathcal {V}= \left( {\begin{smallmatrix} u & \quad v \\ \overline{v} & \quad \overline{u} \end{smallmatrix}} \right) $$ with $v^*v$ having countable spectrum. Let $\overline{\mathscr {F}}$ be the ESS over $\mathcal {E}$ with respect to the basis $(\varvec{g}_k)_{k \in \mathbb {N}} \subset \ell ^2$. Define the new vacuum vector

137 $$\begin{aligned} \Omega _\mathcal {V}= \underbrace{ \exp \left( \frac{1}{4} \sum _k \log \left( 1 - \tfrac{\nu _k^2}{\mu _k^2} \right) \right) }_{=: e^\mathfrak {r}} \underbrace{ \exp {\left( -\sum _k \frac{\nu _k}{2 \mu _k} (a^\dagger (\varvec{g}_k))^2 \right) } }_{=: \Psi _\mathcal {V}} \Omega = e^\mathfrak {r}\Psi _\mathcal {V}\;, \end{aligned}$$

where $\mu _k, \nu _k$ are the singular values of *u*, *v* as in Sect. 3.2. Then, $\mathcal {V}$ is implemented in the sense of (77) by $\mathbb {U}_\mathcal {V}: \mathcal {D}_\mathscr {F}\rightarrow \overline{\mathscr {F}}$ (79).

#### Proposition B.4

(Implementation via ESS works, fermionic) Consider a fermionic Bogoliubov transformation $$ \mathcal {V}= \left( {\begin{smallmatrix} u &  v \\ \overline{v} &  \overline{u} \end{smallmatrix}} \right) $$ with $v^*v$ having countable spectrum. Let $\overline{\mathscr {F}}$ be the ESS over $\mathcal {E}$ with respect to the basis $(\varvec{\eta }_j)_{j \in J}$, and let $|J''_1| < \infty$, so **the number of modes with a full particle–hole transformation is finite**. Define the new vacuum vector

138 $$\begin{aligned} \begin{aligned} \Omega _\mathcal {V}= \underbrace{ \exp \left( \sum _{i \in I'} \log \alpha _i \right) }_{=: e^\mathfrak {r}} \underbrace{ \left( \prod _{j \in J''_1}^\otimes a^\dagger (\varvec{\eta }_j) \right) \left( \prod _{i \in I'}^\otimes \left( 1 - \frac{\beta _i}{\alpha _i} a^\dagger (\varvec{\eta }_{2i}) a^\dagger (\varvec{\eta }_{2i-1}) \right) \right) }_{=: \Psi _\mathcal {V}} \Omega = e^\mathfrak {r}\Psi _\mathcal {V}\;, \end{aligned} \end{aligned}$$

with $\alpha _i, \beta _i$ being the singular values of *u*, *v* as in (40). Then, $\mathcal {V}$ is implemented in the sense of (77) by $\mathbb {U}_\mathcal {V}: \mathcal {D}_\mathscr {F}\rightarrow \overline{\mathscr {F}}$ (79).

In contrast to Theorem 5.6, the additional condition of having finitely many modes with a particle–hole transformation comes from the requirement that $\Omega _\mathcal {V}$ be in $\overline{\mathscr {F}}$.

Diagonalizability of Hamiltonians in an extended sense can then be defined for $\overline{\mathscr {F}}$ exactly as for $\widehat{\mathscr {H}}$ (Definition 6.1). Finally, also Propositions 6.3 and 6.4 for diagonalizability carry over from $\widehat{\mathscr {H}}$ to $\overline{\mathscr {F}}$, with the restriction to finitely many particle–hole transformed modes in the fermionic case.

## Footnotes

1 In relativistic QFT, Haag’s theorem forbids a common representation for free and interacting dynamics [43, Sect. II.1] But such a common representation may also fail to exist in more general situations, like in polaron models [33] or when taking the thermodynamic limit of many-body systems [10, Chapter 17].

2 Here, for $\mathcal {A}_-$, the term “generated” comprises taking the closure.

3 See also the formulation [47, Thm. 10.10] of the spectral theorem.
